# Synthesis and Characterization of a Minophosphonate Containing Chitosan Polymer Derivatives: Investigations of Cytotoxic Activity and in Silico Study of SARS-CoV-19

**DOI:** 10.3390/polym13071046

**Published:** 2021-03-26

**Authors:** Ponnusamy Packialakshmi, Perumal Gobinath, Daoud Ali, Saud Alarifi, Norah Salem Alsaiari, Akbar Idhayadhulla, Radhakrishnan Surendrakumar

**Affiliations:** 1 PG & Research, Department of Chemistry, Nehru Memorial College, Affiliated Bharathidasan University, Puthanamapatti, Tamilnadu 621007, India; packiyaponnusamy96@gmail.com (P.P.); gopi10207005@gmail.com (P.G.); idhayadhulla@nmc.ac.in (A.I.); 2 Department of Zoology, College of Sciences, King Saud University (KSU), P.O. Box 2455, Riyadh 11451, Saudi Arabia; aalidaoud@ksu.edu.sa (D.A.); salarifi@ksu.edu.sa (S.A.); 3 Department of Chemistry, College of Science, Princess Nourahbint Abdulrahman Univerity, Riyadh 11451, Saudi Arabia; nsalsaiari@pnu.edu.sa

**Keywords:** chitosan, Schiff base, spectral analysis, X-ray diffraction, TGA, DSC and cytotoxic activity, In silico analysis

## Abstract

Chitosan is broadly used as a biological material since of its excellent biological activities. This work describes investigations of chitosan interaction with SARS-CoV-2, which is occupied by human respiratory epithelial cells through communication with the human angiotension-converting enzyme II (ACE2). The β-chitosan derivatives are synthesized and characterized by FT-IR, nuclear magnetic resonance (^1^H and ^13^C NMR), mass spectrometry, X-ray diffraction, TGA, DSC, and elemental analysis. The β-chitosan derivatives were screened for cytotoxic activity against the HepG2 and MCF-7 (breast) cancer cell lines. Compound **1**h (GI_50_ 0.02 µM) is moderately active against the HepG2 cancer cell line, and Compound **1**c is highly active (GI_50_ 0.01 µM) against the MCF-7 cancer cell line. In addition, chitosan derivatives (**1**a–**1**j) docking against the SARS coronavirus are found by in-silico docking analysis. The findings show that compound **1**c exhibits notable inhibition ability compared with other compounds, with a binding energy value of −7.9 kcal/mol. Based on the molecular docking results, the chitosan analog is proposed to be an alternative antiviral agent for SARS-CoV2.

## 1. Introduction

**Figure 1:**
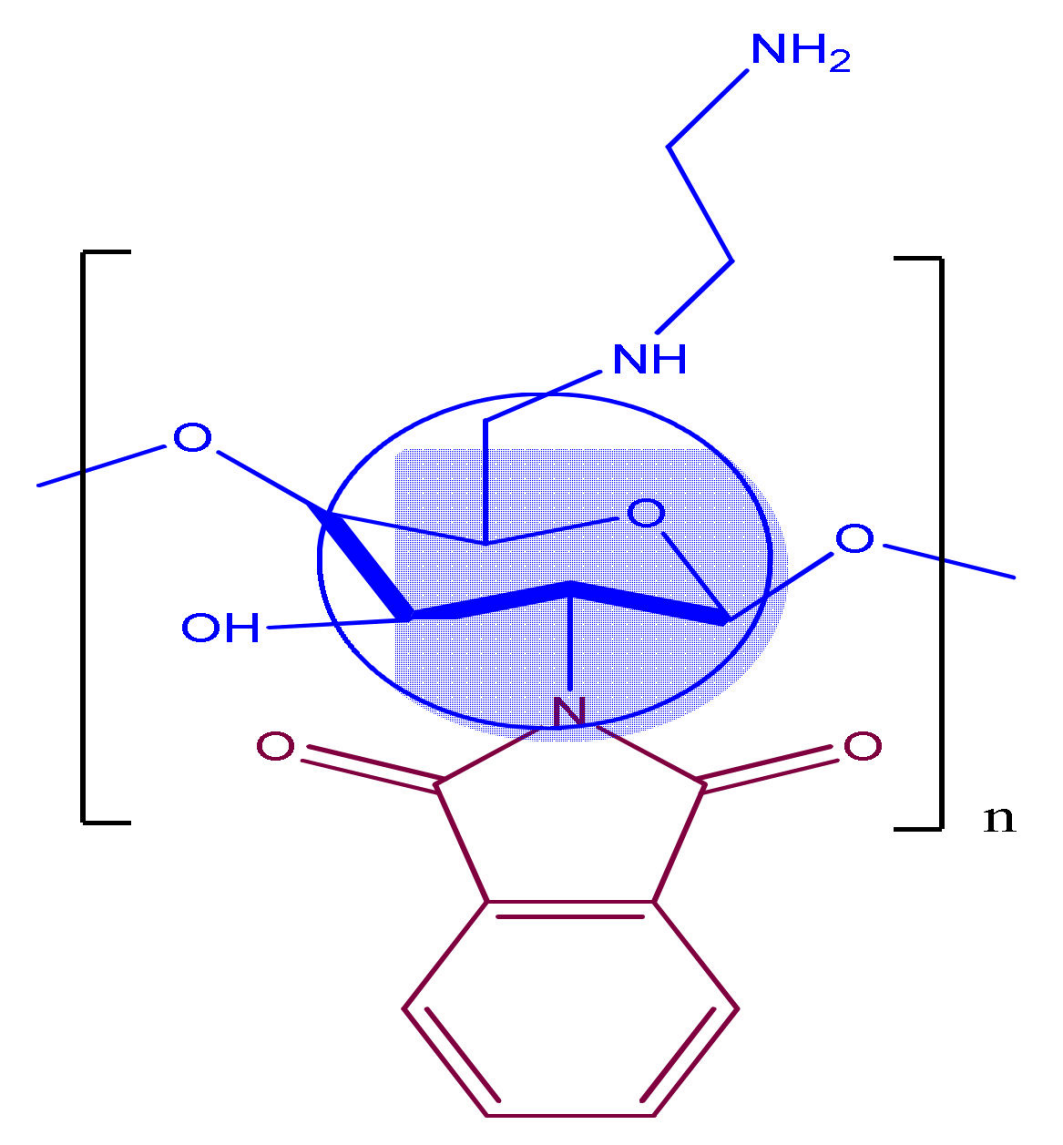
Antiviral activity (Newcastle virus) of chitosan derivatives.

The reported SARS that turned into COVID-19 was originally a coronavirus, SARS-CoV-2. The first cases of SARS-CoV were reported as occurring in November 2002 in China (although not recognized at that time), and the WHO became aware of these in early 2003 [1]. Severe Acute Respiratory Syndrome (SARS) is a novel viral typical pneumonia. This communicable illness was reported on 31 December 2019. The World Health Organization (WHO) was officially informed about cases of pneumonia in Wuhan City, home to 11 million people and the cultural and financial hub of Central China, and afterward quickly extended to 25 other countries [2]. The infection spreads mainly from one person to another through small droplets from the nose or mouth. On 11 August 2020, Russia became the first country in the world to approve a vaccine (Sputnik V) against (SARS-CoV2). The vaccine, which is based on two adenovirus vectors, wasdeveloped by the Gamaleya National Center of Epidemiology and Microbiology (Moscow, Russia) [3], even though steroids and ribavirin have been used in hospitals of Hong Kong [4,5] and HIV protease inhibitor nelfinavir can reduce replication of SARS-CoV based on an initialin vitroassessment [6]. In view of the fact that the SARS CoV 3C-like protease playsa very significant role in the viral life cycle, there has been a key goal planned for the structure-based drug proposal against SARS [7]. The drug-resistant and insufficient antiviral drugs have increased demand for the development of more potent and less toxic antiviral agents. Chitosan-based antiviral research was published as shown in Figure 1 [8]. The chemical alteration of chitosan (CS) has recently been paid attention byresearchers due to its wide variety of applications.

**Figure 2:**
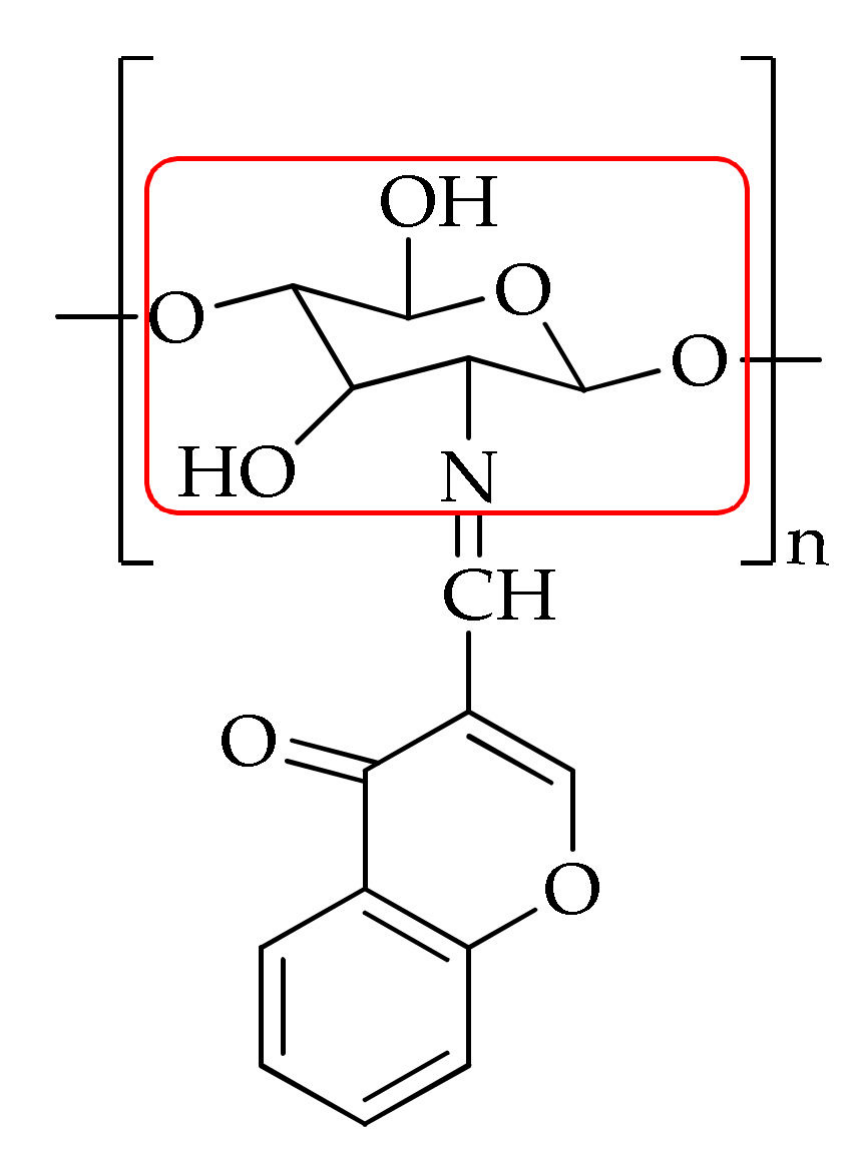
Polymeric structure of chitosan derivatives.

The chemical compositions of chitosan components powerfully influence its biological activity. These factors certainly must be taken into account in an analysis of its antiviral activity [9]. Chitin has a chemical structure of a characteristic supplementary biopolymer found in the exoskeletons of roaches and crustaceans [10], but chitosan is frequently obtained by the deacetylation of chitin [11]. CS has a few receptive NH_2_groups that offer additional chemical alteration for the expansion of derivatives that are cost-effective [12]. Deacetylated chitosan is an extremely useful and readily available bioactive polymer [13,14]. Chitosan displays several beneficial biological properties, such as antitumor and antimicrobial [15]. The antiviral activity of chitosan is determined by its structure and, by the polymerization degree of the molecule [16]. Thus, the high polymeric preparations of polysaccharides possess antiviral activity. On the other hand, it has been shown that the high polymeric chitosan’s significantly increased its antiviral activity [17,18]. It consists of unbranched chain of β-glucose as repeating units [19]. As a result, data on the dependence of chitosan derivative, mainly the polymerization degree, are in significant disagreement, which can be determined by the variety of chitosan sources [20]. Chitosan is depolymerized by chemical hydrolysis, which is performed for obtainingsamples of the polysaccharide. The initial high molecular (HM) samples of chitosan and the methods of its purification also can increase the biological activity of its chitosan derivatives [21]. The polymeric structure of chitosan derivative is given in Figure 2 [22].

**Figure 3:**
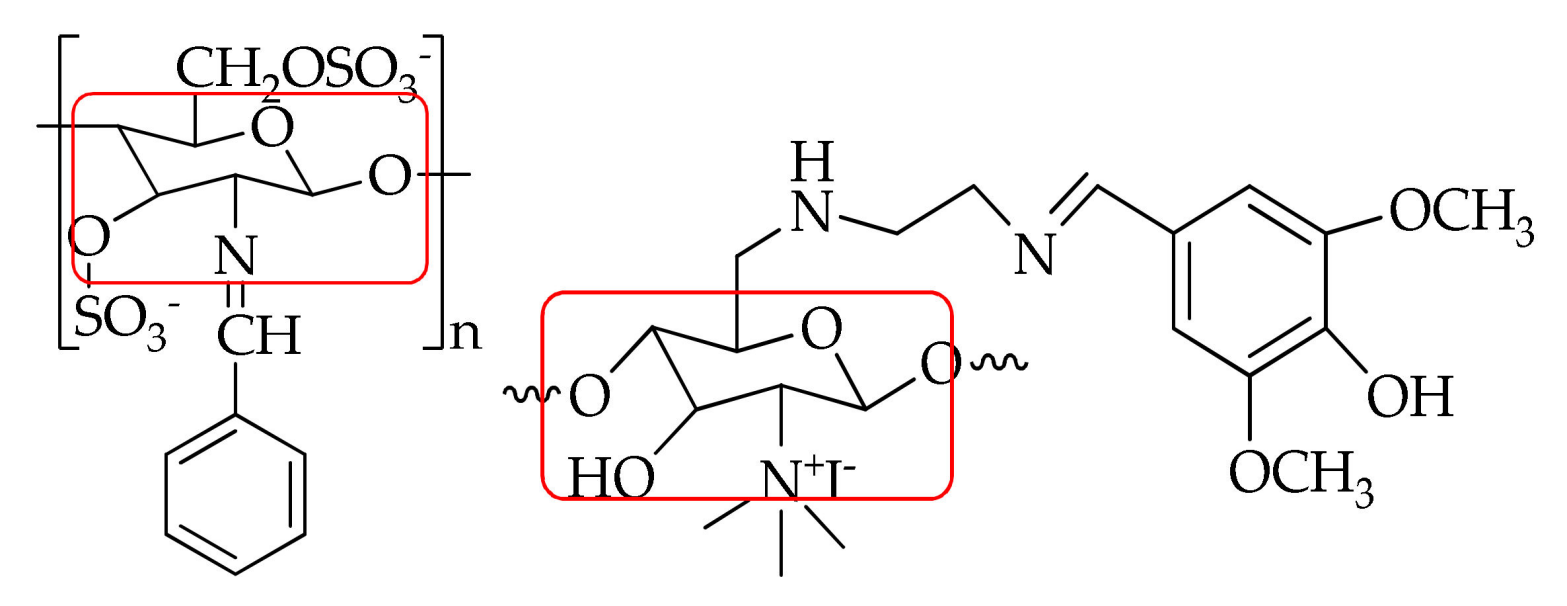
Anticancer activity of chitosan derivatives.

Phosphorylated chitin (P-chitin) and chitosan (P-chitosan) were prepared by the reaction of chitin or chitosan with orthophosphoric acid and urea in DMF [23] with phosphorous pentoxide in methane sulphonic acid [24]. The amino phosphonates are among the most common organophosphorus derivatives and have been used inantiviral, antibacterial, antifungal, and anticancer activities [25,26,27,28,29,30]. Many of the Schiff base chitosan derivatives exhibit pharmacological properties likeantimicrobial and anticancer shown in Figure 3 [31,32].

In the current work, new aminophosphonates containing chitosan moieties are synthesized. Particularly, we graft fluorobenzaldehyde, fluoroaniline, and triphenylphosphite in the presence of lithium perchlorate as a catalyst. To the best of our knowledge, no work on a-aminophosphonate and fluoro aniline with β-chitosan has been previously reported in the literature.

## 2. Experimental

### 2.1. Materials and Methods (Chemistry)

Melting points were recorded in open capillary tubes. The FT-IR spectra wererecorded the KBr disk technique on a Shimadzu 8201pc (4000–1000 cm^−1^). The ^1^H-NMR and ^13^C-NMR spectra were recorded on a Bruker DRX-300 MHz. The compound was dissolved in DMSO-d_6_ to obtain NMR spectra. Mass spectra were recorded by Perkin Elmer GCMS model Clarus SQ8 (EI). The elemental analysis (C, H, and N) was performed using an Elemental analyser model (Varian EL III). The purity of the compounds was checked by TLC with silica gel plates. The starting materials β-chitosan, substituted aldehydes, and fluoro aniline were attained from Sigma Aldrich, India.

#### 2.1.1. Preparation of Chitosan Analogue (**1**a–**1**j)

A mixture containing Compound 1 (0.001 mol, 0.547 g) and 4-fluoroailine (0.01 mol, 0.947 mL) along with 15 mL ethanol and NaOH was added withcontinuous stirring at high temperature, and also underwent dehydration to obtain Compound **1**a as a white solid. The obtained product was treated with ice-cold water. The progress of the reaction was monitored by TLC. Hexane and ethylacetate (4:6) were used as eluting solvents in TLC. Thin-layer chromatography (TLC) was done by CCM Gel de silica gel 60 F254 sheet (0.1 mm) with hexane/ethylacetate (4:6 by volume) as emerging eluents. The compounds were detected passing an E-Series UV hand lamp (254/365 nm wave length). The compounds are purified by column chromatography (gel filtration method, wet condition), and silica gel (60–120 mesh) was used as a sorbent. Hexane/ethyl acetate was used an eluting solvents. The obtained solid was further purified by recrystallization from ethanol. The above procedure was followed for the synthesis of Compounds **1**b–**1**j.

Synthesis of diphenylN-(4-fluorophenyl)-P-((1)-(4-fluorophenyl) (((2,3,6)-4-hydroxyl-6-(hydroxylmethyl)-2,5-dimethoxytetrahydro-2H-pyran-3-yl)amino)methyl)phosphonimidate (**1**a) White solid; yield 60%; mp 108 °C; IR (kBr) (cm^−1^); 3450 (OH, str), 3280 (NH-str), 3050 (CH-str Ar ring), 2925 (CH, str), 1425 (N-C, str), 1120 (P=N), 890–900 (P-O-C, str). ^1^H NMR (CDCl_3_), δ (ppm): 7.95 (s, 1H, NH), 7.90–7.21 (m, 4H, F-Ph), 7.28–7.01 (m, 10H, PO-(Ph)_3_), 6.85–6.63 (m, 4H, F-aniline), 5.83 (s, 2H, CS-OH), 5.56 (d, 1H, CH, *J* = 14–17 Hz), 4.87 (m, 5H, CS-H), 3.50 (d, 2H, CS, *J* = 10 Hz), 3.52 (s, 6H, CS).^13^C NMR (CDCl_3_) δ (ppm):161.4, 116.8, 115.9 (6C, F-Ph), 157.7, 155.3, 142.5, 115.3 (6C, F-Ph-NH_2_), 152.7, 124.9, 121.5, 121.3 (12C, PO-(Ph)_3_), 102.8, 85.6, 76.3, 73.1, 67.6, 60.5, 59.5, 55.7 (8C, CS), 75.6 (1C, CH). EI-MS (relative intensity %): *m*/*z* 642.22 (M^+^, 20%). Elemental analysis: Anal.C_33_H_35_F_2_N_2_O_7_P: C, 61.87; H, 5.51; N, 4.35; Found, C, 61.88; H, 5.50; N, 4.37.

Diphnyl-P-((1)-(4-chlorophenyl)(((2,3,6)-4-hydroxy-6-(hydroxymethyl)-2,5-dimethoxytetrahdro-2H-pyran-3-yl)amino)methyl)-N-(4-fluorophenyl)phosphonimidate (**1**b) Light yellow solid; yield 70%; mp120 °C; IR (kBr) (cm^−1^); 3420 (OH, str), 3250 (NH-str), 3030 (CH-str Ar ring), 2935 (CH, str), 1372 (N-C, str), 1120 (P=N), 900 (P-O-C, str). ^1^H NMR (CDCl_3_), δ (ppm):9.28 (s, 1H, NH), 7.95–7.52 (m, 4H, Cl-Ph), 7.26–7.00 (m, 10H, PO-(Ph)_3_), 6.83–6.60 (m, 4H, F-aniline), 5.85 (s, 2H, CS-OH), 5.62 (d, 1H, CH, *J* =15 Hz), 4.85 (m, 5H, CS-H), 3.52 (d, 2H, CS, *J* = 10 Hz), 3.50 (s, 6H, CS).^13^C NMR (CDCl_3_) δ (ppm):157.5, 155.6, 142.3, 115.7 (6C, F-Ph-NH_2_), 152.4, 124.5, 121.7, 121.6 (12C, PO-(Ph)_3_), 135.4, 125.3, 120.4, 116.5 (6C, Cl-Ph), 102.3, 85.5, 76.7, 73.4, 67.2, 60.1, 59.6, 55.5 (8C, CS), 75.3 (1C, CH). EI-MS (relative intensity %): *m*/*z* 658.18 (M^+^, 21%). Elemental analysis: Anal.C_33_H_35_ClFN_2_O_7_P: C, 60.32; H, 5.32; N, 4.26; Found, C, 60.34; H, 5.33; N, 4.25.

DiphenylN-(4-fluorophenyl)-P-((1)-(((2,3,6)-4-hydroxy-6-(hydroxymethyl)-2,5-dimethoxytetrahydro-2H-pyran-3-yl)amino)(4-hydroxyphenyl)methyl)phosphonimidate (**1**c) Light yellow powder; yield 78%; mp 138 °C; IR (kBr)(cm^−1^); 3405 (OH, str), 3252 (NH-str), 3033 (CH-str Ar ring), 2937 (CH, str), 1372 (N-C, str), 1120 (P=N), 905 (P-O-C, str). ^1^H NMR (CDCl_3_), δ (ppm): 10.60 (s, 1H, Ph-OH), 9.34 (s, 1H, NH), 7.75, (m, 4H, OH-Ph), 7.24–6.98 (m, 10H, PO-(Ph)_3_), 6.87 (m, 4H, F-aniline), 5.82 (s, 2H, CS-OH), 5.58 (d, 1H, CH, *J* = 15 Hz), 4.80–3.85 (m, 5H, CS-H), 3.55 (d, 2H, CS, *J* = 10 Hz), 3.53 (s, 6H, CS).^13^C NMR (CDCl_3_) δ (ppm):161.6, 129.9, 120.3, 116.0 (6C, OH-Ph), 157.4, 155.7, 142.4, 115.8 (6C, F-Ph-NH_2_), 152.6, 124.7, 121.8, 121.4 (12C, PO-(Ph)_3_), 102.4, 85.6, 76.8, 73.5, 67.2, 60.3, 59.7, 55.3 (8C, CS), 75.1 (1C,CH). EI-MS (relative intensity %): *m*/*z* 640.23 (M^+^,19%). Elemental analysis: Anal.C_33_H_36_FN_2_O_8_P: C, 62.06; H, 5.68; N, 4.39; Found, C, 62.07; H, 5.69; N, 4.40.

DiphenylN-(4-fluorophenyl)-P-((1)-(((2,3,6)-4-hydroxy-6-(hydroxymethyl)-2,5-dimethoxytetrahydro-2H-pyran-3-yl)amino)(4methoxyphenyl)methyl)phosphonimidate (**1**d) White solid; yield 65%; mp 146 °C; IR (kBr) (cm^−1^); 3435 (OH, str), 3225 (NH-str), 3038 (CH-str Ar ring), 2931 (CH, str), 1378 (N-C, str), 1173 (P=N), 903 (P-O-C, str). ^1^H NMR (CDCl_3_), δ (ppm):9.25 (s, 1H, NH), 7.85–6.99 (m, 4H, OCH_3_-Ph), 7.07–6.93 (m, 10H, PO-(Ph)_3_), 6.89–6.63 (m, 4H, F-aniline), 5.80 (s, 2H, CS-OH), 5.54 (d, 1H, CH, *J* =15 Hz), 4.85–3.82 (m, 5H, CS-H), 3.85 (s, 3H, OCH_3_), 3.59 (d, 2H, CS, *J* = 10 Hz), 3.30 (s, 6H, CS).^13^C NMR (CDCl_3_) δ (ppm):160.6, 130.2, 124.3, 114.5 (6C, OCH_3_-Ph), 157.0, 155.4, 142.5, 115.9 (6C, F-Ph-NH_2_), 152.5, 124.6, 121.7, 121.3 (12C, PO-(Ph)_3_), 102.7, 85.5, 76.3, 73.1, 67.4, 60.1, 59.8, 55.2 (8C, CS), 75.0 (1C, CH), 55.4 (1C, OCH_3_). EI-MS (relative intensity %): *m*/*z* 654.24 (M^+^, 23%). Elemental analysis: Anal.C_34_H_38_FN_2_O_8_P: C, 62.57; H, 5.87; N, 4.29; Found, C, 62.59; H, 5.89; N, 4.30.

DiphenylP-((1)-(4-(dimethylamino)phenyl)(((2,3,6)-4-hydroxy-6-(hydroxymethyl)-2,5-dimethoxytetrahydro-2H-pyran-3-yl)amino)methyl)-N-(4fluorophenyl)phosphonimidate (**1**e) White solid; yield 80%; mp 153 °C; IR (kBr) (cm^−1^); 3430 (OH, str), 3227 (NH-str), 3034 (CH-str Ar ring), 2936 (CH, str), 1382 (N-C, str), 1176 (P=N), 900 (P-O-C, str). ^1^H NMR (CDCl_3_), δ (ppm):9.28 (s, 1H, NH), 7.68 (m, 4H, N(CH_3_)_2_-Ph), 7.20–7.05 (m, 10H, PO-(Ph)_3_), 6.85–6.61 (m, 4H, F-aniline), 5.82 (s, 2H, CS-OH), 5.53 (d, 1H, CH, *J* =15 Hz), 4.83 (m, 5H, CS-H), 3.59 (d, 2H, CS, *J* =10 Hz), 3.30 (s, 6H, CS), 3.07 (s, 3H, N(CH_3_)_2_).^13^C NMR (CDCl_3_) δ (ppm):157.0, 155.4, 142.5, 115.9 (6C, F-Ph-NH_2_), 152.5, 124.6, 121.7, 121.3 (12C, PO-(Ph)_3_), 151.6, 128.9, 120.8, 111.6, 20.4 (8C, N(CH_3_)_2_-Ph), 102.0, 85.2, 76.4, 73.5, 67.1, 60.4, 59.7, 55.1 (8C, CS), 75.4 (1C,CH). EI-MS (relative intensity %): *m*/*z* 667.27 (M^+^, 21%). Elemental analysis: Anal.C_35_H_41_FN_3_O_7_P: C, 63.15; H, 6.21; N, 6.33; Found, C, 63.17; H, 6.22; N, 6.35.

DiphenylN-(4-fluorophenyl)-P-((1)-(((2,3,6)-4-hydroxy-6-(hydroxymethyl)-2,5-dimethoxytetrahydro-2H-pyran-3-yl)amino)(3-nitrophenyl)methyl)phosphonimidate (**1**f) Light yellow solid; yield 92%; mp 132 °C; IR (kBr) (cm^−1^); 3435 (OH, str), 3226 (NH-str), 3034 (CH-str Ar ring), 2937 (CH, str), 1550 (N-O, str), 1382 (N-C, str), 1175 (P=N), 903 (P-O-C, str). ^1^H NMR (CDCl_3_), δ(ppm): 9.32 (s, 1H, NH), 8.50–8.30 (m, 4H, NO_2_-Ph), 7.18–6.92 (m, 10H, PO-(Ph)_3_), 6.83–6.60 (m, 4H, F-aniline), 5.81 (s, 2H, CS-OH), 5.58 (d, 1H, CH, *J* = 15 Hz), 4.82 (m, 5H, CS-H), 3.54 (d, 2H, CS, *J* =10 Hz), 3.32 (s, 6H, CS).^13^C NMR (CDCl_3_) δ (ppm):157.2, 155.6, 142.4, 115.7 (6C, F-Ph-NH_2_), 152.3, 124.4, 121.6, 121.2 (12C, PO-(Ph)_3_), 148.3, 136.1, 135.3, 130.1, 129.5, 123.6 (6C, NO_2_-Ph), 102.1, 85.3, 76.2, 73.4, 67.3, 60.5, 59.6, 55.2 (8C, CS), 75.8 (1C,CH). EI-MS (relative intensity %): *m*/*z* 669.22 (M^+^, 20%). Elemental analysis: Anal.C_33_H_35_FN_3_O_9_P: C, 59.37; H, 5.28; N, 6.29; Found, C, 59.38; H, 5.29; N, 6.30.

DiphenylN-(4-fluorophenyl)-P-((1)-(((2,3,6)-4-hydroxy-6-(hydroxymethyl)-2,5-dimethoxytetrahydro-2H-pyran-3-yl)amino)(2-hydroxyphenyl)methyl)phosphonimidate (**1**g) White solid; yield 73%; mp 160 °C; IR (kBr) (cm^−1^); 3407 (OH, str), 3225 (NH-str), 3034 (CH-str Ar ring), 2934 (CH, str), 1382 (N-C, str), 1174 (P=N), 907 (P-O-C, str). ^1^H NMR (CDCl_3_), δ (ppm): 9.30 (s, 1H, NH), 7.72–7.21 (m, 4H, OH-Ph), 7.16–6.94 (m, 10H, PO-(Ph)_3_), 6.80–6.62 (m, 4H, F-aniline), 5.80 (s, 2H, CS-OH), 5.59 (d, 1H, CH, *J* = 15 Hz), 5.35 (s, 1H, OH-Ph), 4.83–3.98 (m, 5H, CS-H), 3.52 (d, 2H, CS, *J* = 10 Hz), 3.30 (s, 6H, CS).^13^C NMR (CDCl_3_) δ (ppm): 161.8, 135.9, 131.4, 122.0, 121.5, 118.4 (6C, OH-Ph), 157.3, 155.5, 142.6, 115.3 (6C, F-Ph-NH_2_), 152.4, 124.2, 121.5, 121.3 (12C, PO-(Ph)_3_), 102.2, 85.4, 76.5, 73.7, 67.8, 60.6, 59.4, 55.3 (8C, CS), 75.6 (1C,CH). EI-MS (relative intensity %): *m*/*z* 640.23 (M^+^, 22%). Elemental analysis: Anal.C_33_H_36_FN_2_O_8_P: C, 62.06; H, 5.68; N, 4.39; Found, C, 62.07; H, 5.69; N, 4.40.

DiphenylN-(4-fluorophenyl)-P-((1)-(4-hydroxy-3-methoxyphenyl)(((2,3,6)-4-hydroxy-6-(hydroxylmethyl)-2,5-dimethoxytetrahydro-2H-pyran-3-yl)amino)methyl) phosphonimidate (**1**h) Light yellow solid; yield 86%; mp 140 °C; IR (kBr) (cm^−1^); 3404 (OH, str), 3220 (NH-str), 3034 (CH-str Ar ring), 2932 (CH, str), 1382 (N-C, str), 1175 (P=N), 905 (P-O-C, str). ^1^H NMR (CDCl_3_), δ (ppm):9.79 (s, 1H, OH), 9.28 (s, 1H, NH), 7.42–6.75 (m, 3H, vanillin), 7.14–7.06 (m, 10H, PO-(Ph)_3_), 6.82–6.65 (m, 4H, F-aniline), 5.83 (s, 2H, CS-OH), 5.57 (d, 1H, CH, *J* = 15 Hz), 4.70 (m, 5H, CS-H), 3.84 (s, 3H, OCH_3_), 3.55 (d, 2H, CS, *J* =10 Hz), 3.34 (s, 6H, CS).^13^C NMR (CDCl_3_) δ (ppm): 157.3, 155.5, 142.6, 115.3 (6C, F-Ph-NH_2_), 154.5, 149.6, 130.4, 125.2, 117.4, 110.5 (6C, vanillin), 152.6, 124.3, 121.2, 121.4 (12C, PO-(Ph)_3_), 102.5, 85.3, 76.6, 73.8, 67.4, 60.5, 59.1, 55.2 (8C, CS), 75.3 (1C, CH), 56.2 (1C, OCH_3_). EI-MS (relative intensity %): *m*/*z* 670.24 (M^+^,19%). Elemental analysis: Anal.C_34_H_38_FN_2_O_9_P: C, 51.07; H, 5.73; N, 4.19; Found, C, 51.09; H, 5.74; N, 4.20.

DiphenylN-(4-fluorophenyl)-P-((1)-(((2,3,6)-4-hydroxy-6-(hydroxymethyl)-2,5-dimethoxytetrahydro-2H-pyran-3-yl)amino)-3-phenylallyl)phosphonimidate (**1**i) White solid; yield 79%; mp 165 °C; IR (kBr) (cm^−1^); 3445 (OH, str), 3223 (NH-str), 3034 (CH-str Ar ring), 2936 (CH, str), 1383 (N-C, str), 1172 (P=N), 896 (P-O-C,str).^1^H NMR (CDCl_3_), δ (ppm): 9.29 (s, 1H, NH), 8.13–7.60 (m, 7H, Ph), 7.12–6.94 (m, 10H, PO-(Ph)_3_), 6.84–6.63 (m, 4H, F-aniline), 5.85 (s, 2H, CS-OH), 5.60 (d, 1H, CH, *J* = 17 Hz), 4.66 (m, 5H, CS-H), 3.58 (d, 2H, CS, *J* =10 Hz), 3.30 (s, 6H, CS).^13^C NMR (CDCl_3_) δ (ppm): 157.5, 155.3, 142.7, 115.2 (6C, F-Ph-NH_2_), 152.6, 135.2, 128.9, 128.6, 128.3, 127.9 (8C, Ph), 152.2, 124.6, 121.7, 121.5 (12C, PO-(Ph)_3_), 102.7, 85.5, 76.3, 73.6, 67.2, 60.3, 59.8, 55.4 (8C, CS), 75.4 (1C, CH). EI-MS (relative intensity %): *m*/*z* 650.25 (M^+^,20%). Elemental analysis: Anal.C_35_H_38_FN_2_O_7_P: C, 64.81; H, 5.90; N, 4.32; Found, C, 64.83; H, 5.92; N, 4.34.

DiphenylN-(4-fluorophenyl)-P-((1)-furan-2-yl(((2,3,6)-4-hydroxy-6(hydroxylmethyl)-2,5-dimethoxytetrahydro-2H-pyran-3-yl)amino)methyl)phosphonimidate(**1**j) White solid; yield 80%; mp 143 °C; IR (kBr) (cm^−1^); 3440 (OH, str), 3230 (NH-str), 3036 (CH-str Ar ring), 2932 (CH, str), 1382 (N-C, str), 1175 (P=N), 900 (P-O-C, str). ^1^H NMR (CDCl_3_), δ (ppm):9.29 (s, 1H, NH), 8.09–7.47 (m, 3H, furfural), 7.10–6.92 (m, 10H, PO-(Ph)_3_), 6.82–6.64 (m, 4H, F-aniline), 5.82 (s, 2H, CS-OH), 5.62 (d, 1H, CH, *J* = 17 Hz), 4.60 (m, 5H, CS-H), 3.60 (d, 2H, CS, *J* = 10 Hz), 3.33 (s, 6H, CS).^13^C NMR (CDCl_3_) δ (ppm):157.5, 155.3, 142.7, 115.2 (6C, F-Ph-NH_2_), 153.3, 147.2, 121.5, 112.8 (4C, furfural), 152.4, 124.2, 121.5, 121.7 (12C, PO-(Ph)_3_), 102.6, 85.4, 76.7, 73.2, 67.5, 60.8, 59.1, 55.3 (8C, CS), 75.2 (1C, CH). EI-MS (relative intensity %): *m*/*z* 614.21 (M^+^, 24%). Elemental analysis: Anal.C_31_H_34_FN_2_O_3_P: C, 60.78; H, 5.59; N, 4.57; Found, C, 60.80; H, 5.61; N, 4.58.

#### 2.1.2. Cytotoxic Activity

The newly synthesized compounds (**1**a–**1**j) were screened for their cytotoxic activity according to a procedure described in previous literature [33,34]. Three cancer cell lines were treated with these compoundsat one primary cytotoxic assay dose of 100 µM for 48 h (MTTanticancer assay). Doxorubicin was used as a standard. The two cell lines used in the present investigation were HepG2 (liver) and MCF-7(breast). In the current protocol, all cell lines were pre-incubated on a microtiter plate. The results of each test were reported as the growth percentage of treated cells compared to untreated control cells (Table 1). The screening results were expressed in terms of the growth inhibitor concentration (GI_50_), total growth of inhibition (TGI), and lethal concentration (LC_50_). A 0.1 mL aliquot ofthecell suspension (5 × 10^4^ cells/100 µL) and 0.1 mL ofthetest solution (6.25–100 µg in 1% DMSO, the final DMSO concentration in media less than 1%) were added to the wells, with the plates kept in an incubator (5% CO_2_) at 37 °C for 72 h. The results were analyzed using the statistical software SPSS version 16.0. Manufacturers and suppliers of SPSS software company IBM located in USA, New York, Armonk.

#### 2.1.3. Molecular Docking

Molecular docking tests are regularly used to analyze binding interactions. The relationships of between the target protein (PDB ID: 6LU7) and the synthesized chitosan derivatives **1**a–**1**j were determined with AutodockVina 1.1 [35]. The crystal structure SARS coronavirus core protease (PDB ID: 6LU7) was obtained from the Protein Data Bank (http:/www.rcsb.org (accessed on 5 September 2020)). ChemDraw Ultra 12.0 and Chem3D Pro 12.0 programming suites were utilized to sketch the 3D structures of the ligand molecules and energyminimization. AutoDock Tools 1.5.6 was utilized to fabricate the AutodockVina input contents. In manufacturing the co-crystallized inhibitor molecule using the discovery studio program, α-ketoamide was used to identify the binding pocket. The amino acid residues Thr24, Thr26, Phe140, Asn142, Gly143, Cys145, His163, His164, Glu166, and His172were present in the binding pocket. The SARS COVID-19 main protease quest grid was recognized as center x: −10.656, center y: 17.223, center z: 67.024 in dimension x: 20, y: 20, and z: 20 in 1.0 Å spacing. The significance of completeness was set to 8. The extra limitations were fixed and not indicated by default for Autodock Vina. Thecompound that consumed the smallest binding affinity value is achieved the highest score, the implications of which were visually examined with Discovery studio 2019.

#### 2.1.4. Structure Activity Relationship

**Figure 4:**
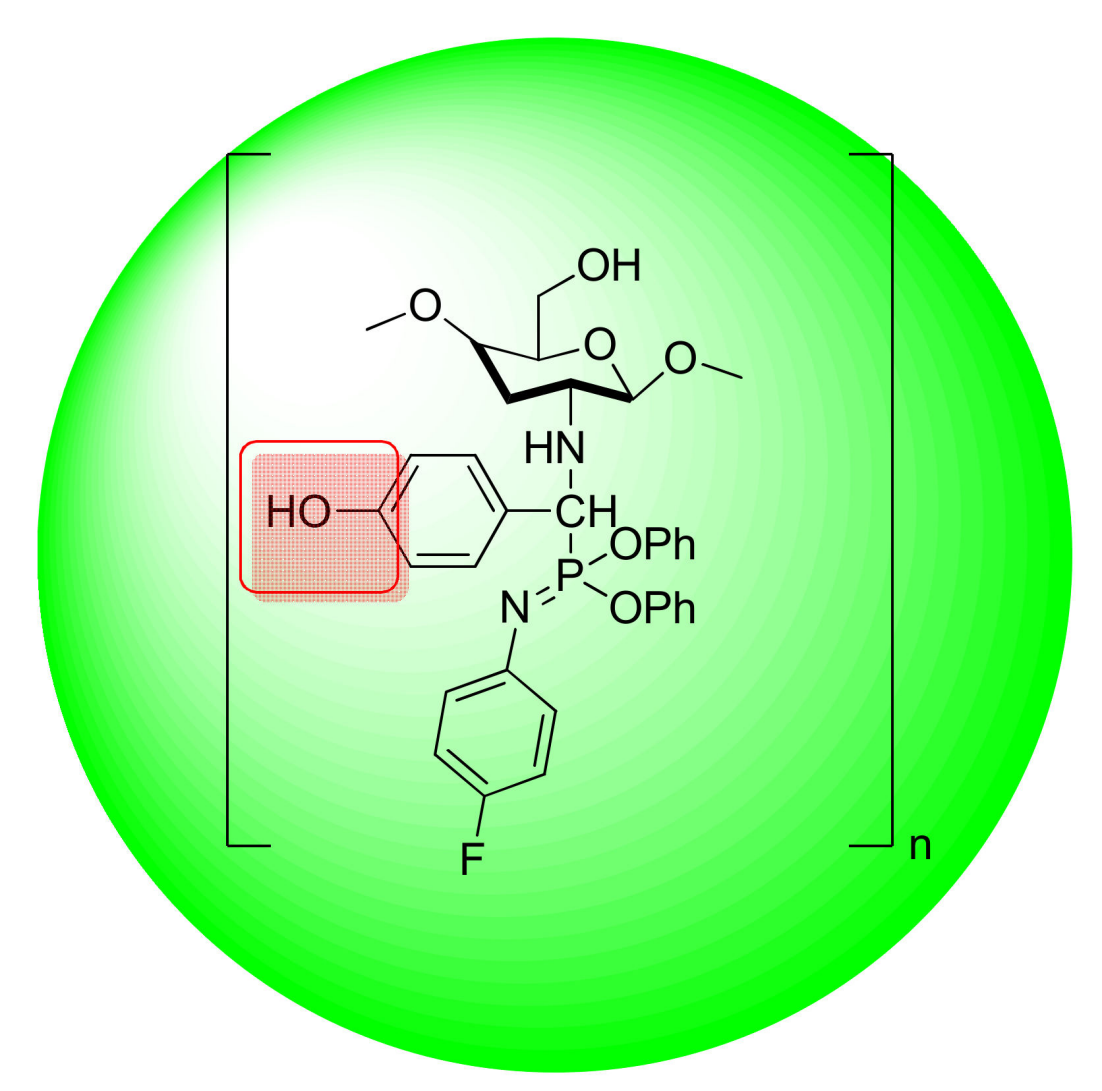
Highly active compound structure.

A structure–activity relationship analysis (SAR) can be used to investigate the relationship between the chemical structure of an active molecule and its biological activity in a specific assay system. SAR analysis makes it possible to identify the chemical group/atom that plays a critical role in modulating the cytotoxic activity of compounds in the specific system. Based on the cytotoxic activity results of thederivatives preliminary SARs could be evaluated. The data of the selected chitosan derivatives (**1**a–**1**j) evidenced that compound **1**c is highly active (GI_50_ 0.01 µM) against the MCF-7 cancer cell linecompared with control Doxorubicin. Compound **1**c docking score −7.9 kcal/mol. Figure 4 indicates highly active compound in cytotoxic activity and molecular docking analysis.

Due to the presence of β-chitosan beingfused to a hydroxy benzaldehyde, it was found that the compound acquires a high cytotoxic activity against cancer cell lines.

The reason would be the presence of electron releasing hydroxyl group on phenyl ring attached with the β-chitosan skeleton. The rest of the compounds showed weak cytotoxic activity against all the tested cancer cell lines. Moreover, from the docking results, it can be assumed that the docking score for β-chitosan derivatives (**1**a–**1**j) has an acceptable range.

## 3. Result and Discussion

**Scheme 1:**
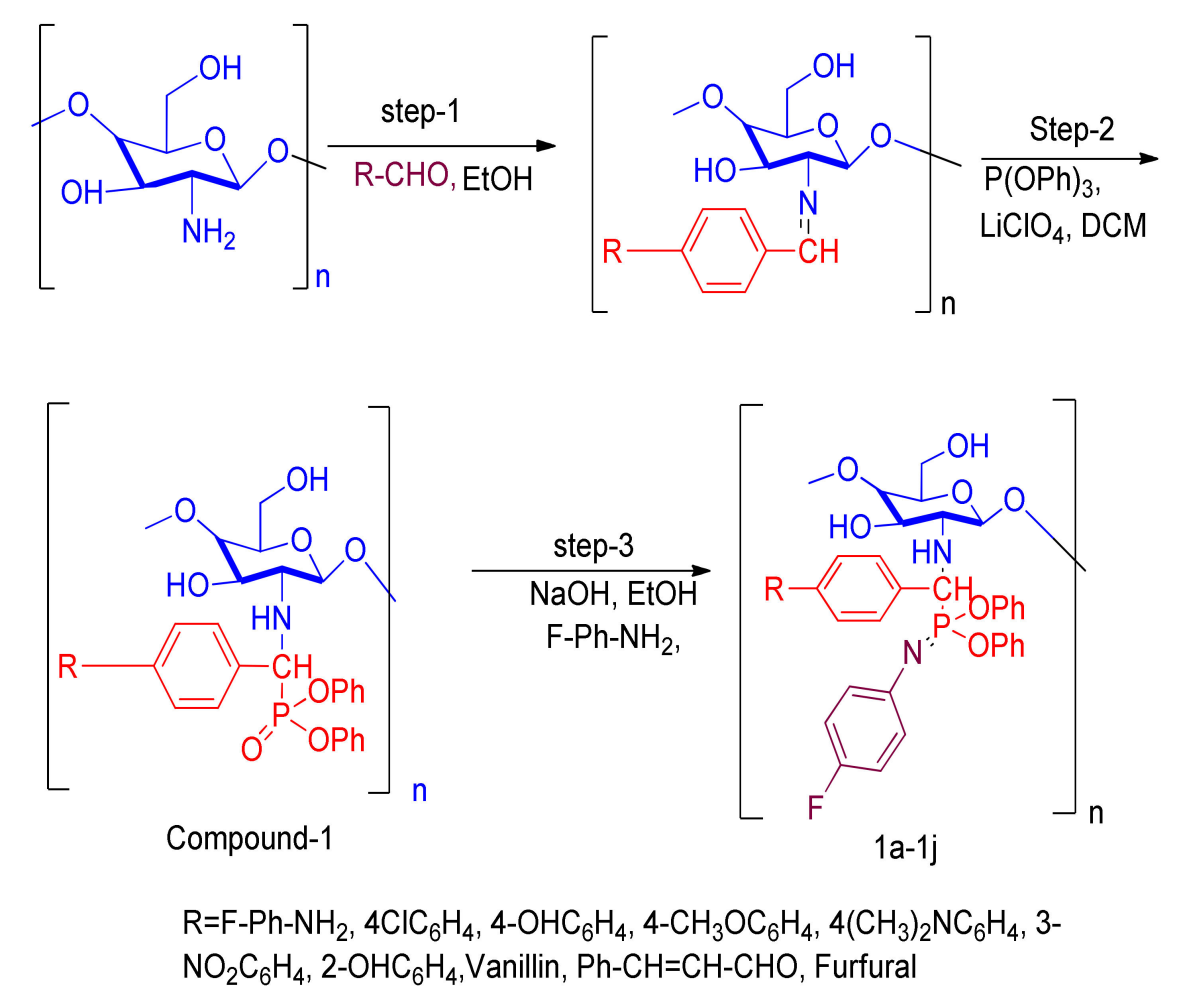
Synthesis of chitosan derivatives.

The chitosan derivatives of compound **1** were prepared following a method found in the literature [36]. Synthesis of amino phosphate chitosan involved Schiff base formation and fluoro aniline was added to β-chitosan (Mwt.577) (**1**a–**1**j) by the procedure described in [37]. The amino group present in β-chitosan (C2-Position) are significant because they are nucleophilic and eagerly react with electrophilic reagents. Because of this, biologically active moieties were grafted into the amino group of chitosan. Compound **1** reacted with 4-fluoroaniline, NaOH, and ethanol added at high temperature and underwent a dehydration reaction to obtain Compound **1**a as a white solid. The reaction was monitored by thin layer chromatography (TLC). After completion of the reaction, the crude product was recrystallized by ethanol. The above procedure was followed for other (**1**b–**1**j) synthesized chitosan derivatives. The synthesized compounds were confirmed by analytical technique, such as FT-IR, ^1^H NMR and ^13^C NMR as seen in Supplementary Materials (Figures S1–S3). The FT-IR spectra exhibited absorption bands (**1**a–**1**j) at 895, 1170, 1383, 3045, 3250, and 3450cm^−1^, confirming P-O-C, P=N, N-C, Ar-H, NH, and OH functional groups. The ^1^H NMR spectra (1a–1j) showed chemical shift values at δ 10.60, 9.34–7.95, 8.50–6.64, 7.28–6.96, 6.87–6.60, 5.55, and 3.85 ppm, confirmingOH, NH, P-O-Ph, F-NH_2_, CH, and OCH_3_ protons, respectively. The ^13^C NMR showed (**1**b–**1**j) signals at157.7–115.3, 152.7–121.3, 75.3, and 56.2 ppm, confirming the F-NH_2_, P-O-Ph, CH, and OCH_3_ of carbon atoms. Chitosan derivatives were affirmed by mass spectroscopy and elemental analysis. The synthesis of chitosan derivatives are shown in Scheme 1.

### 3.1. XRD (or) X-ray Diffraction Study

**Figure 5:**
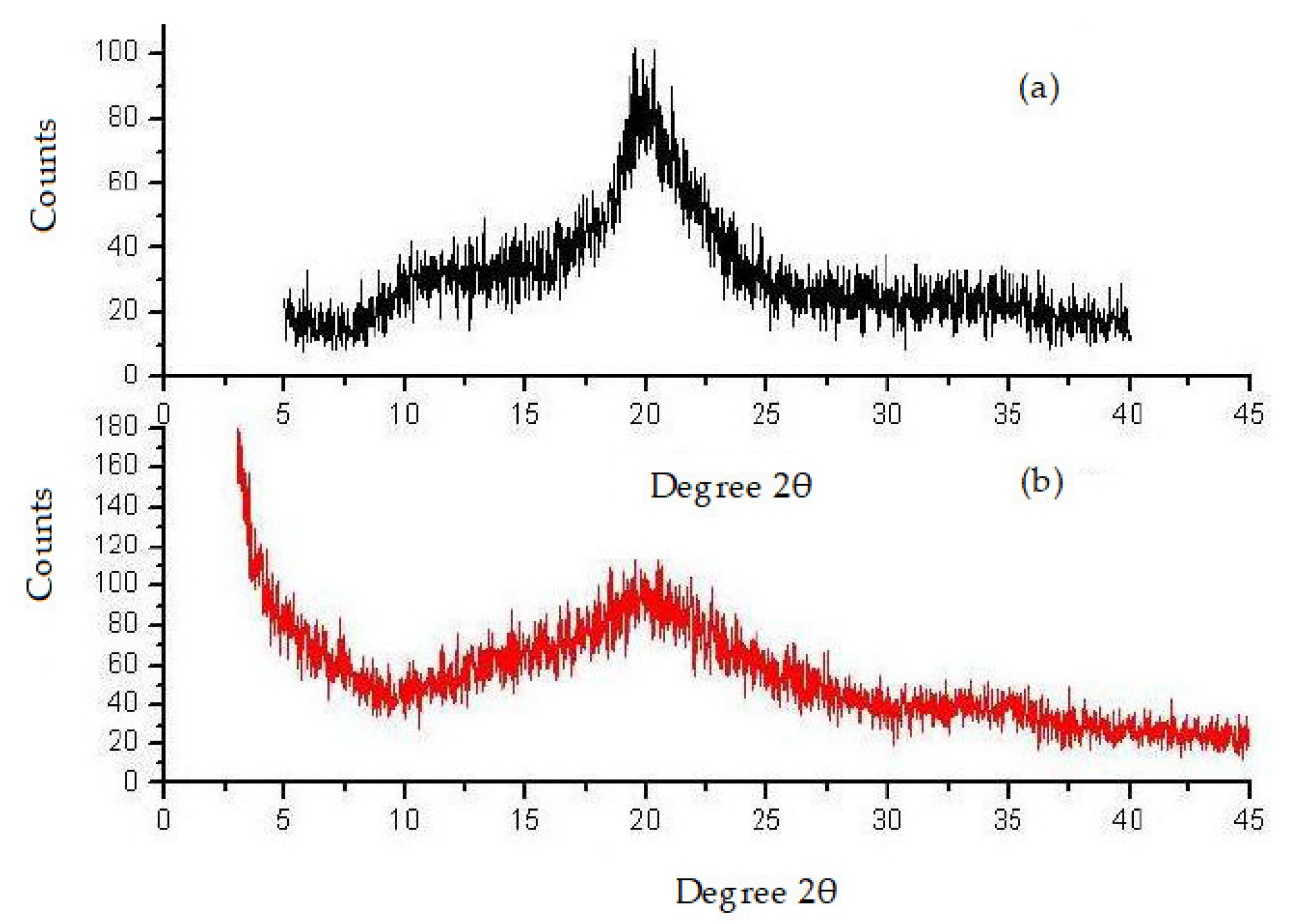
X-ray Diffraction Study of pure chitosan (**a**) and chitosan derivative (**b**).

The chitosan and chitosan derivatives were carried out in an X-Ray diffraction study. Chitosan has observed with very broad peaks at 2θ = 10° and 2θ = 20° Figure 5 [38]. The chitosan derivatives showed two weak peaks at 2θ of 21° and 38° Figure 5. The peak detected for chitosan at 2θ = 10° disappeared and the very broad peaks at 20° became weak in chitosan derivatives, it shows that chitosan has moral compatibility and good formation of porous xerogel network. The XRD result indicates that chitosan derivatives are an amorphous form.

#### 3.1.1. Thermal Analysis of TGA

**Figure 6:**
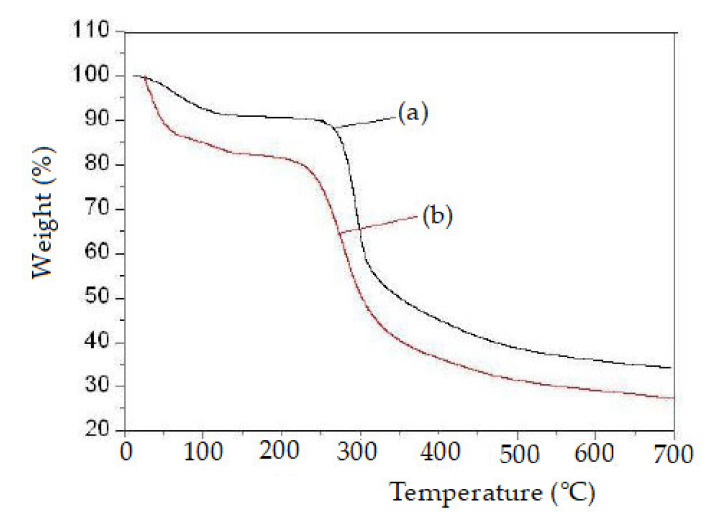
Thermogravimetric analysisof pure chitosan (**a**) and chitosan derivative (**b**).

Figure 6 shows TGA thermograms of pure chitosan and chitosan derivatives. The weight loss of pure chitosan occurs in two stages at the range of 50 to 455 °C. The first occurs in the range of 50–100 °C due to loss of H_2_O molecule with weight loss of 10%. The pure chitosan primary degradation starts at 250 °C, and the entire pure chitosan at 455 °C with a weight loss of 35% [38]. TGA of chitosan derivatives displayed two stages of weight loss in Figure 6. Initial weight loss of chitosan derivative starting from 31 °C to 92 °C may represent a loss of adsorbed H_2_O molecule. The next (second) stage decomposition occurs in the range of 230–410 °C, due to heat decomposition with a weight loss of 51%. The above result describes the loss of thermal stability for chitosan derivatives compared with pure chitosan.

#### 3.1.2. Thermal Analysis of DSC

**Figure 7:**
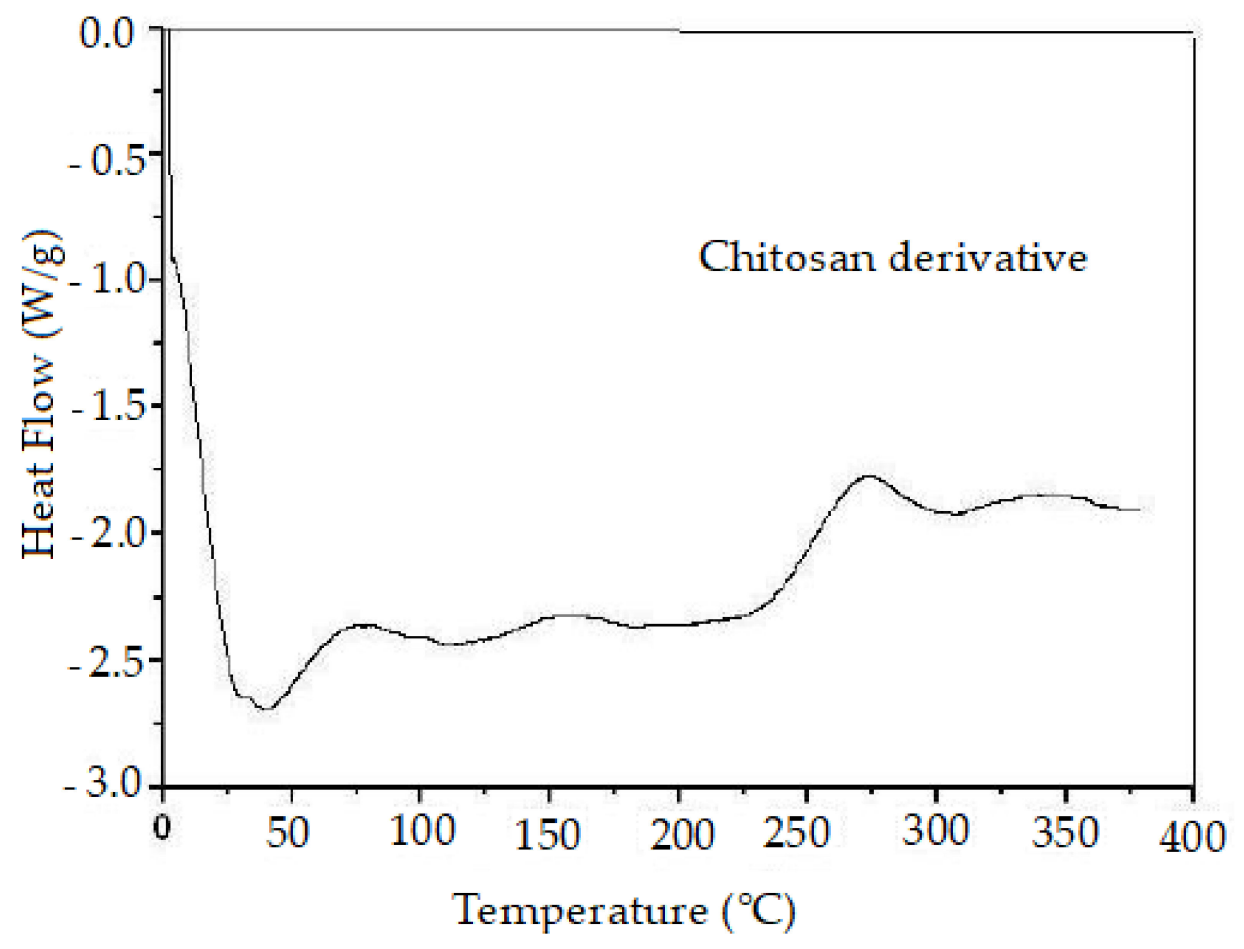
Differential scanning calorimetry of chitosan derivative.

The DSC thermogram of chitosan derivatives is given in Figure 7. Figure 7 indicates the sharp endothermic peaks at 95 °C due to the loss of H_2_O molecules. Then the thermal decomposition of the chitosan derivative occurs in one exothermic peak at 298 °C. Finally, the structures of the chitosan chain have changed due to the fluoro aniline (or) triphenylphosphite and also have a reduced ability to crystalize.

#### 3.1.3. Cytotoxicity Screening

The compounds were tested for cytotoxicity against the HepG2 and MCF-7 cancer cell lines. Compound **1**h showed moderate activity against HepG2 (GI_50_ 0.02 µM), and compound **1**c is highly active (GI_50_ 0.01 µM) against the MCF-7 cancer cell line. All other compounds exhibited lower activity compared to doxorubicin. Values are given in Table 1.

#### 3.1.4. Docking Studies

**Figure 8:**
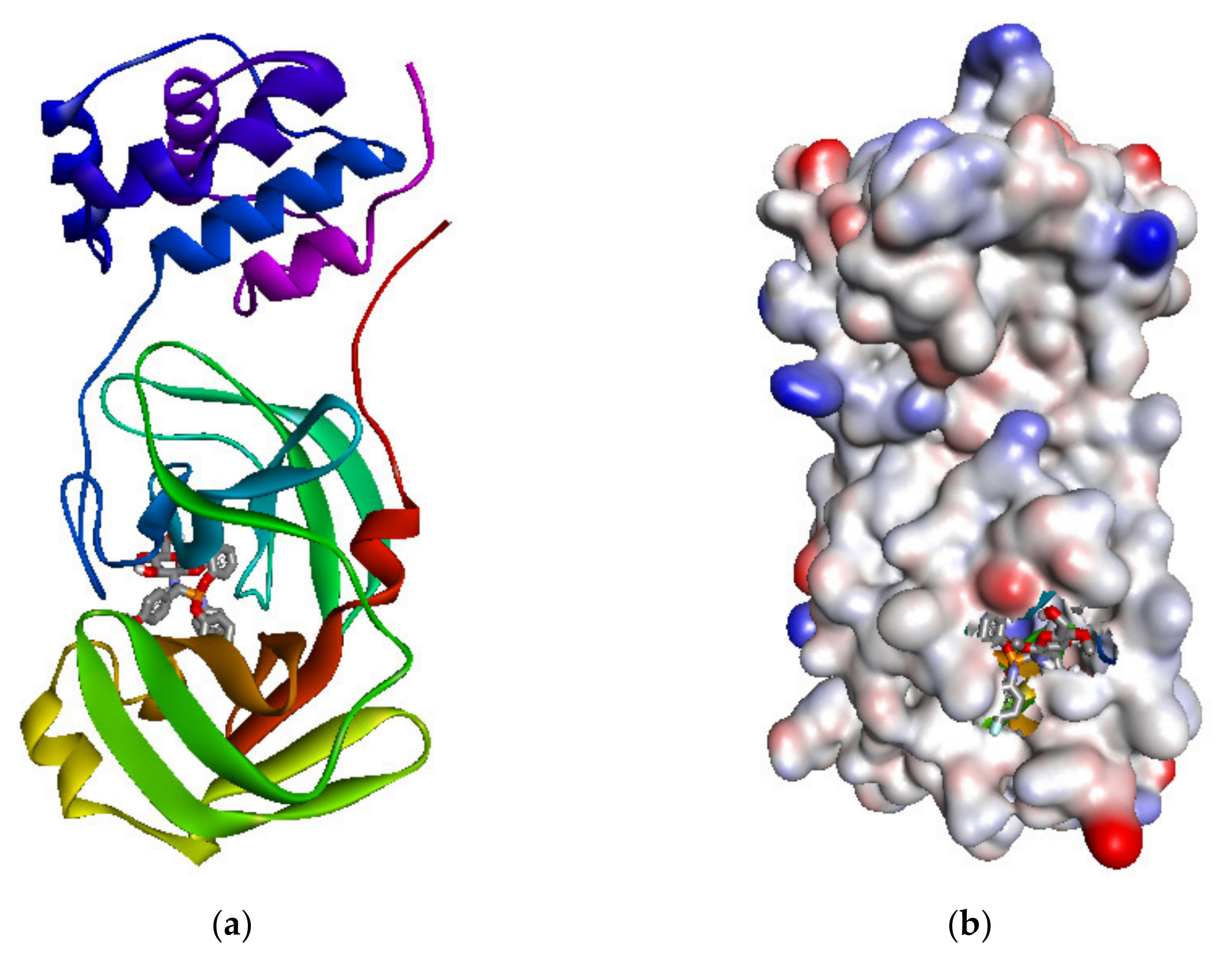

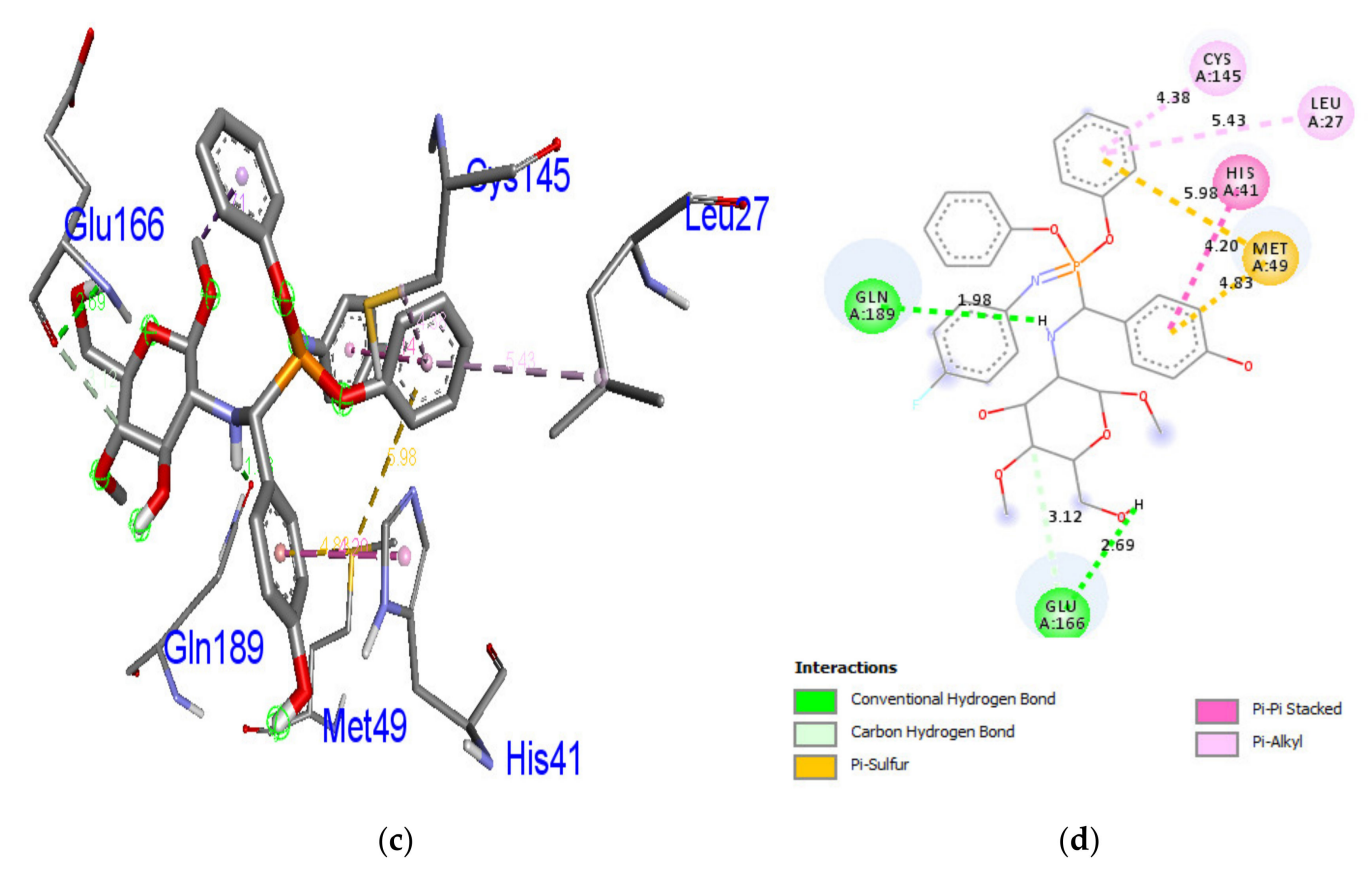
Helix (**a**), molecular surface (**b**), 3D (**c**), and 2D (**d**) interaction modes of compound **1c** within the binding site of. 6LU7 protein.

Docking stimulations were carried out in order to foster understanding of the possible biological activity processes. The compounds (**1**a–**1**j) were analyzed by AutodockVina for their docking behavior with the SARS corona virus main protease (PDB ID: 6LU7). The studied inhibitors show negative binding energy. Compound **1**c demonstrates a remarkable inhibition ability with a binding energy value of −7.9 kcal/mol, compared with compounds **1**a (−7.7 kcal/mol), **1**b (−7.3 kcal/mol), **1**d (−7.8 kcal/mol), **1**e (−7.4 kcal/mol), **1**f (−7.8 kcal/mol), **1**g (−7.4 kcal/mol), **1**h (−7.2 kcal/mol), **1**i (−7.8 kcal/mol), and **1**j (−7.5 kcal/mol) in the target protein correspondingly.Hydrogen bonding serves a significant function in ligand binding stability,and the approvable bond gap between H-acceptor and H-donor atoms is less than 3.5 Å [39]. The gap between the hydrogen bound compounds in the target protein 6LU7 was less than 3.5 Å, which means that the protein and ligands were involved in a resilient hydrogen bond interaction. Compound **1**c displays two hydrogen bonding associations with the 6LU7 receptor. The amino acid residues of Glu166 and Gln189 were entangled through the relationship of the hydrogen linkage with bond lengths between 2.69 and 1.98 Å. The amino acid residues of Leu27, His41, Met49, and Cys145 were in hydrophobic contact. Figure 8 reveals the hydrogen bonding and hydrophobic contacts in the 6LU7 protein of the amino acid residue with compound **1**c. The findings reveal that compound **1**c has an excellent capacity to inhibit the SARS corona virus main protease (PDB ID: 6LU7) compared with other compounds. The findings are presented in Table 2.

## 4. Conclusions

Newly synthesized chitosan analogs (**1**a–**1**j) were confirmed by spectroscopic, X-ray diffraction, TGA, and DSC techniques and screened forin-silico molecular docking analysis against the SARS coronavirus main protease (PDB ID: 6LU7). Compound **1**c exhibits excellent binding affinity −7.9 kcal/mol against the target protein (PDB ID: 6LU7). Compound **1**h (GI_50_ 0.02 µM) is moderately active against the HepG2 cancer cell line, and Compound **1**c is highly active (GI_50_ 0.01 µM) against the MCF-7 cancer cell line. The synthesized chitosan derivative of compound **1**c is a potential antiviral drug and isconsidered a potential therapeutic molecule for COVID-19.

## Figures and Tables

**Table 1:** Cytotoxic effect of compounds (µM) **1**a–**1**j.

Compounds	HepG2			MCF-7		
	GI_50_ (µM)	TGI (µM)	LC_50_ (µM)	GI_50_ (µM)	TGI (µM)	LC_50_ (µM)
**1**a	5.1 ± 0.39	10.1 ± 0.26	19.2 ± 0.15	2.9 ± 0.21	4.2 ± 0.20	10.2 ± 0.02
**1**b	3.3 ± 0.24	7.2 ± 0.54	16.2 ± 0.38	0.23 ± 0.30	5.0 ± 0.74	18.2 ± 0.12
**1**c	0.07 ± 0.09	3.1 ± 0.82	6.2 ± 0.08	0.01 ± 0.28	0.18 ± 0.31	0.20 ± 0.05
**1**d	0.09 ± 0.14	0.12 ± 0.20	0.25 ± 0.18	5.9 ± 0.14	10.5 ± 0.48	19.2 ± 0.11
**1**e	4.9 ± 0.20	9.2 ± 0.23	17.2 ± 0.12	1.9 ± 0.98	7.0 ± 0.34	14.2 ± 0.16
**1**f	3.1 ± 0.32	6.9 ± 0.21	12.2 ± 0.10	2.2 ± 0.71	4.3 ± 0.13	8.2 ± 0.10
**1**g	1.4 ± 0.11	2.8 ± 0.41	18.2 ± 0.12	1.5 ± 0.38	6.3 ± 0.13	16.2 ± 0.65
**1**h	0.02 ± 0.20	0.14 ± 0.85	0.57 ± 0.12	0.04 ± 0.29	0.31 ± 0.62	0.75 ± 0.12
**1**i	7.9 ± 0.33	14.9 ± 0.45	26.9 ± 0.91	2.7 ± 0.27	7.9 ± 0.17	12.3 ± 0.19
**1**j	4.8 ± 0.21	8.8 ± 0.87	16.9 ± 0.57	4.2 ± 0.39	8.4 ± 0.84	20.3 ± 0.98
Doxorubicin	0.01 ± 0.20	0.13 ± 0.14	0.58 ± 0.08	0.02 ± 0.52	0.21 ± 0.84	0.74 ± 0.20

Data represent the mean ± standard deviation (SD) of the mean values of three separate experiments.

**Table 2:** Molecular docking interaction of compounds (**1**a–**1**j) against protease of SARS coronavirus in complex with α-ketoamide (PDB ID: 6LU7).

Compounds	Main Protease of SARS Coronavirus (PDB ID: 6LU7)		
	Binding Affinity (kcal/mol)	No. of H-Bonds	H-Bonding Residues
(**1**a)	−7.7	2	Glu166, Gln189
(**1**b)	−7.3	2	Gly143, Gln189
(**1**c)	−7.9	2	Glu166, Gln189
(**1**d)	−7.8	2	Glu166, Gln189
(**1**e)	−7.4	2	Asn142, Gln189
(**1**f)	−7.8	0	-
(**1**g)	−7.4	1	Gly143
(**1**h)	−7.2	2	Gly143, Gln189
(**1**i)	−7.8	1	Asn142, Gln189
(**1**j)	−7.5	1	Cys145

## Data Availability

Data available in Supplementary Materials.

## Supplementary Materials

The following are available online at https://www.mdpi.com/article/10.3390/polym13071046/s1, Figure S1. FT-IR spectra of compound **1**a–**1**j. Figure S2. 1H-NMR spectra of compound **1**a–**1**j. Figure S3. 13C-NMR spectra of compound **1**a–**1**j.
