# Supplementary material for: Synthesis and Characterization of a Minophosphonate Containing Chitosan Polymer Derivatives: Investigations of Cytotoxic Activity and in Silico Study of SARS-CoV-19
Source: Polymers (Basel). 2021 Mar 26;13(7):1046. doi: 10.3390/polym13071046 (PMC8038110; doi:10.3390/polym13071046)
Supplement: Supplementary file 1 [file polymers-13-01046-s001.pdf]

polymers

MDPI

Supplementary Materials: Synthesis and Characterization of Aminophosphonate Containing Chitosan Polymer Derivatives: Investigations of Cytotoxic Activity and *In Silico* Study of SARS-CoV-19

Ponnusamy Packialakshmi, Perumal Gobinath, Daoud Ali, Saud Alarifi, Norah Salem Alsaiari, Akbar Idhaya-dhulla and Radhakrishnan Surendrakumar

Contents:

FT-IR Spectra of compounds2-11

$^{1.}$ *H-NMR Spectrum of compounds*

$^{13.}$ *C-NMR Spectrum of the compounds22-31*

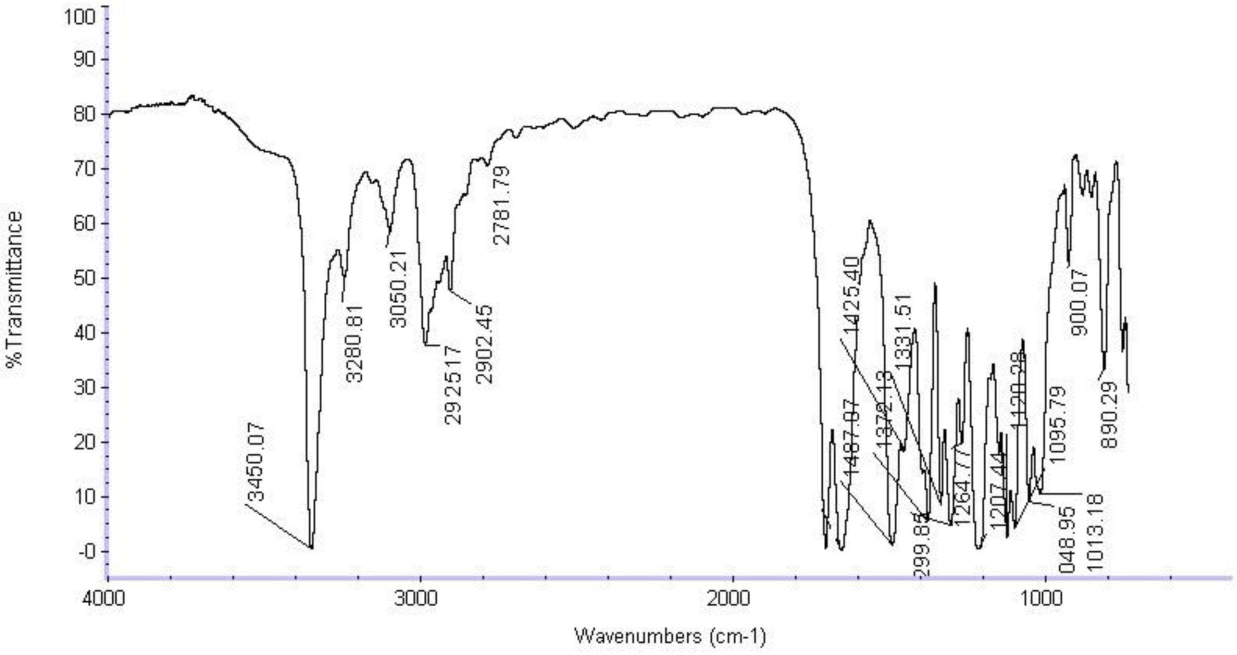

Figure S1. FT-IR spectra of compound-1a.

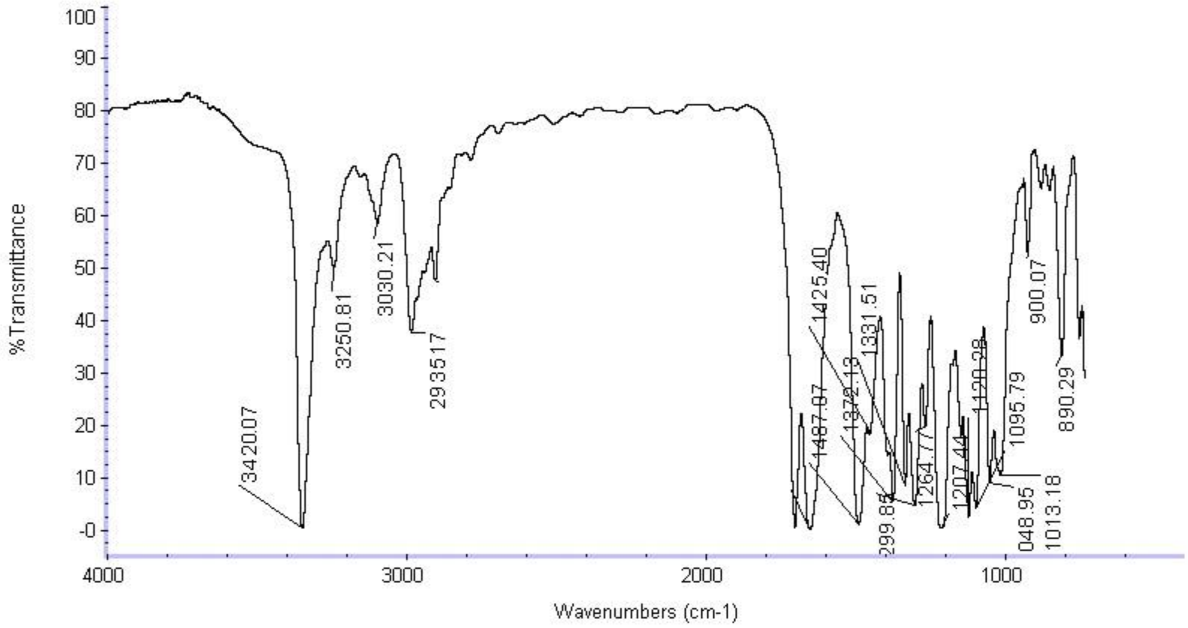

Figure S2. FT-IR spectra of compound-1b.

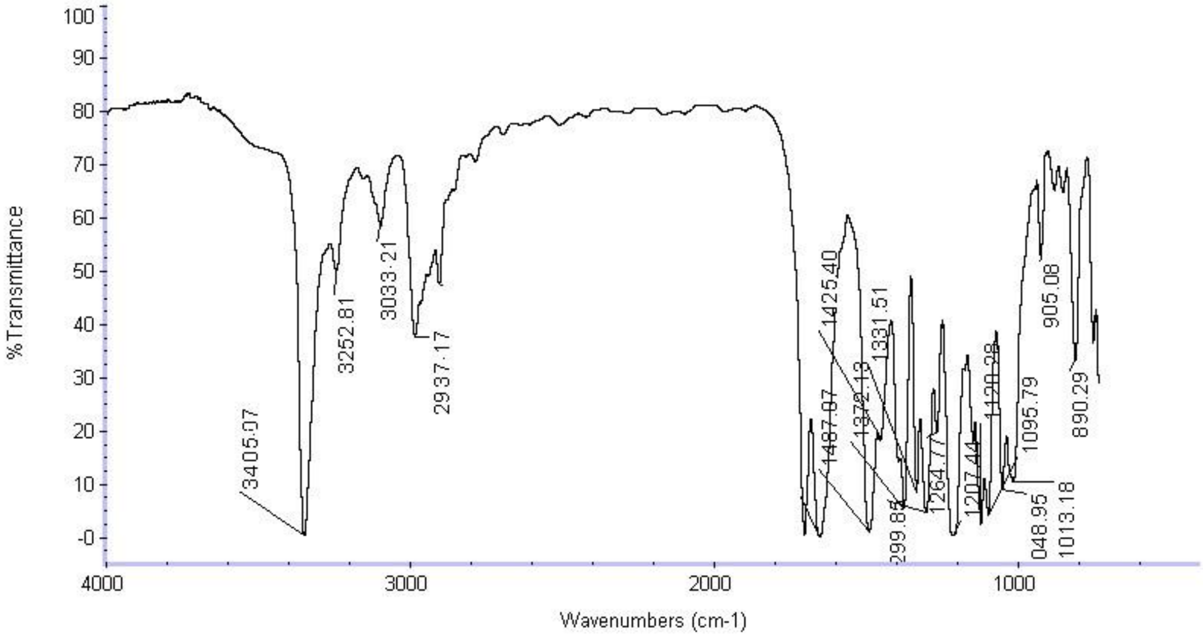

Figure S3. FT-IR spectra of compound-1c.

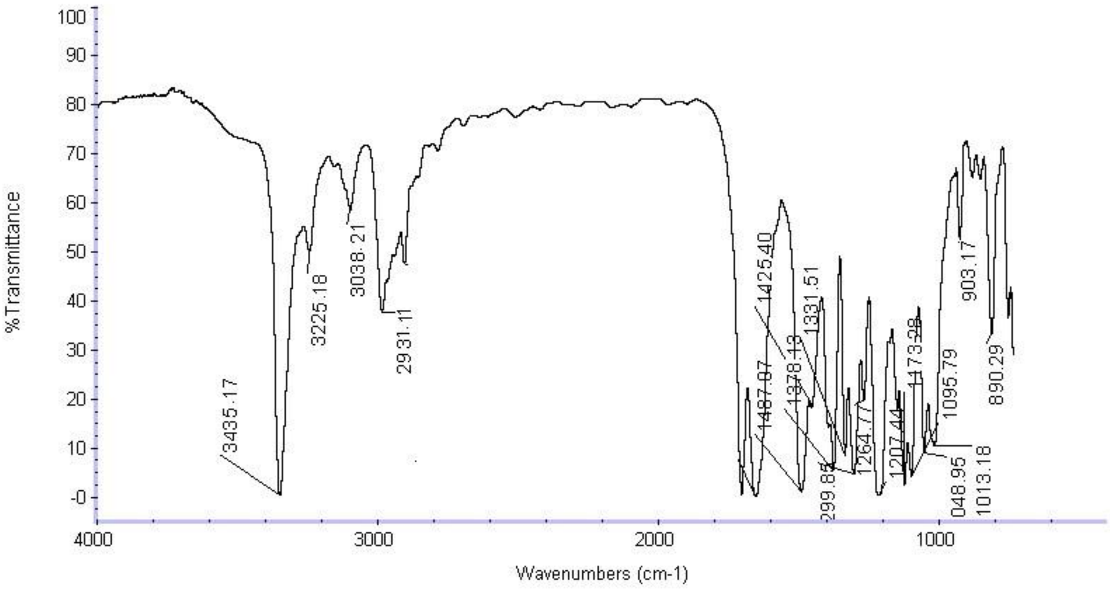

Figure S4. FT-IR spectra of compound-1d.

Compound-1e

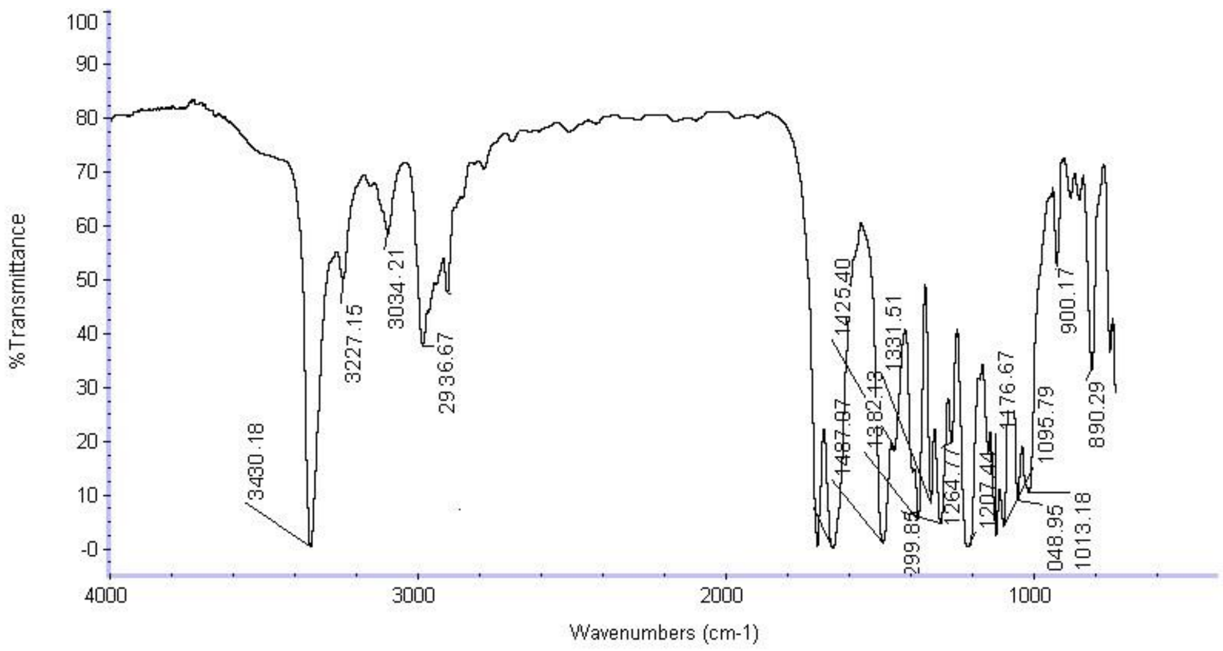
%Transmittance
Wavenumbers (cm-1)
3430.18
3227.15
3034.21
2936.67
1425.40
1487.07
1382.13
1331.51
1299.86
1264.77
1207.44
1176.67
1095.79
1048.95
1013.18
900.17
890.29

Compound-1f

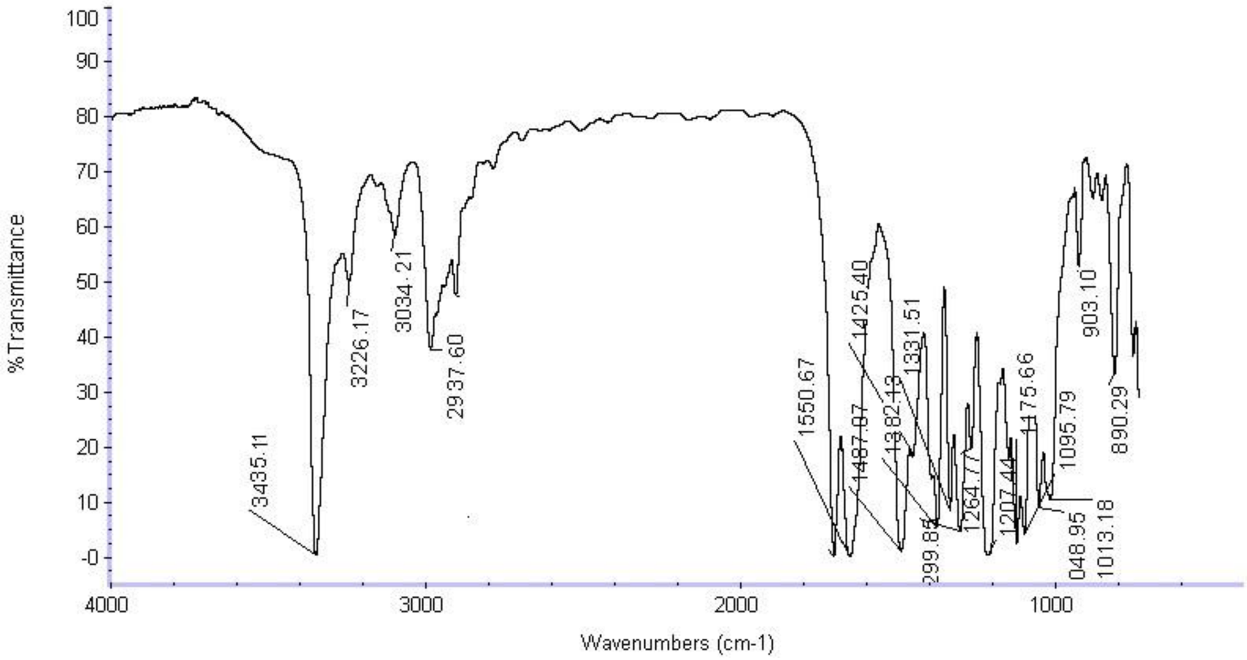
%Transmittance
100
90
80
70
60
50
40
30
20
10
-0
4000
3000
2000
1000
Wavenumbers (cm-1)
3435.11
3226.17
3034.21
2937.60
1550.67
1425.40
1487.87
1382.13
1331.51
299.85
1264.77
1207.44
1175.66
1095.79
048.95
1013.18
903.10
890.29

Compound-1g

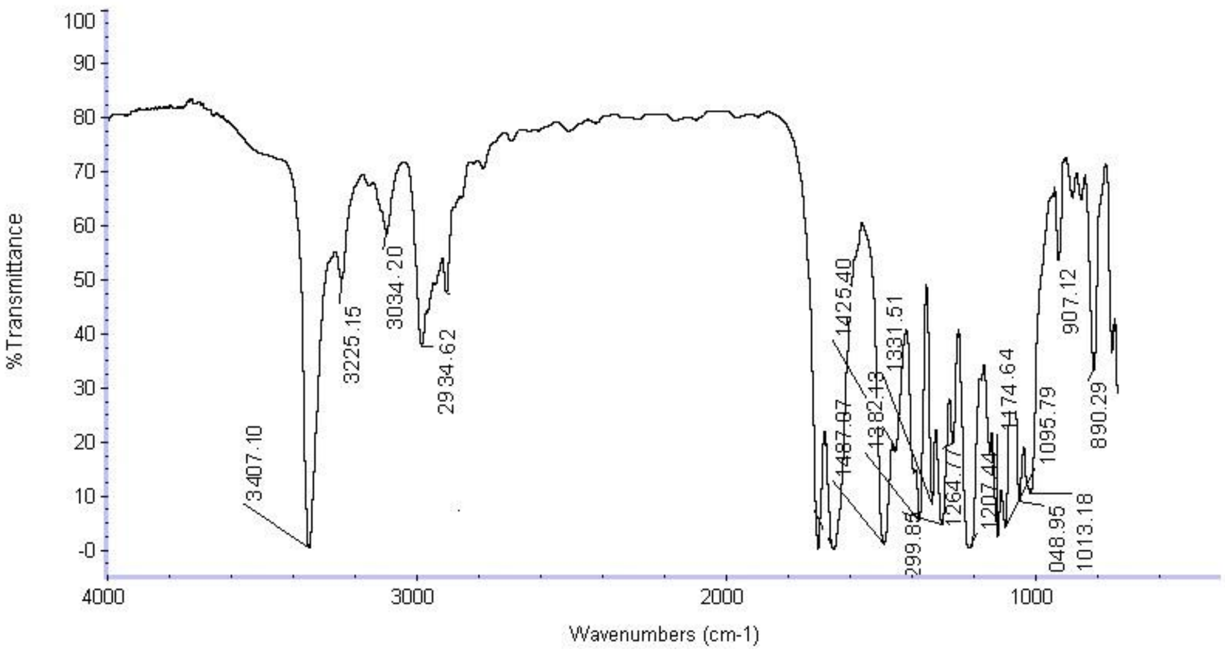

%Transmittance
Wavenumbers (cm-1)
100
90
80
70
60
50
40
30
20
10
-0
4000
3000
2000
1000
3407.10
3225.15
3034.20
2934.62
1425.40
1331.51
1487.87
1382.13
1264.77
1207.44
1174.64
1095.79
1013.18
907.12
890.29

Compound-1h

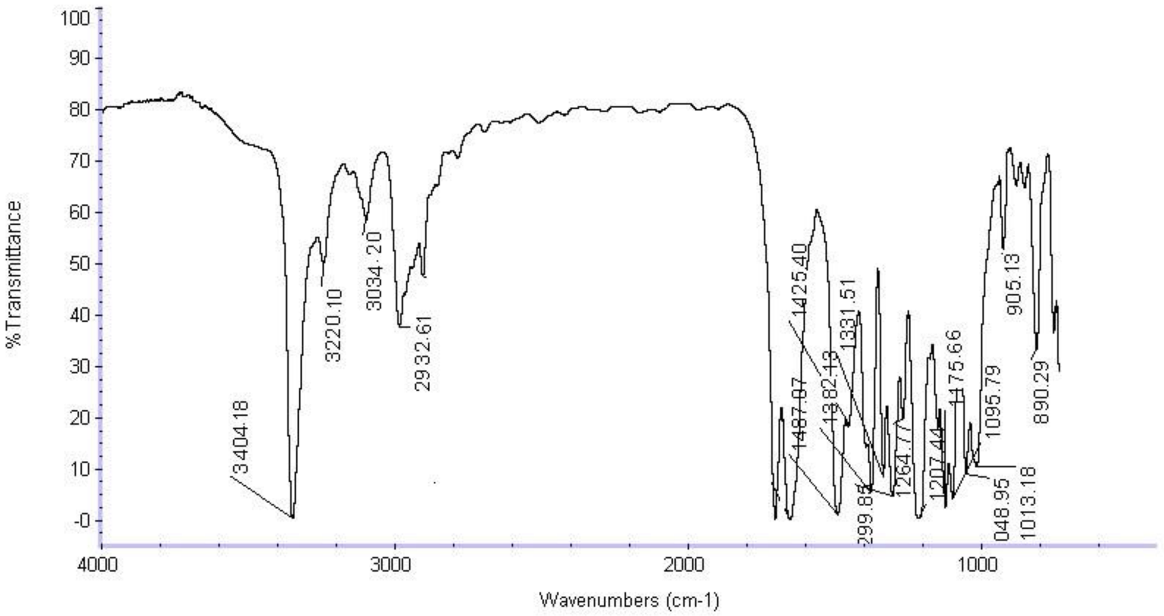
%Transmittance
Wavenumbers (cm-1)
100
90
80
70
60
50
40
30
20
10
-0
4000
3000
2000
1000
3404.18
3220.10
3034.20
2932.61
1487.87
1425.40
1382.13
1331.51
1264.77
1207.44
1175.66
1095.79
1013.18
905.13
890.29

Compound-1i

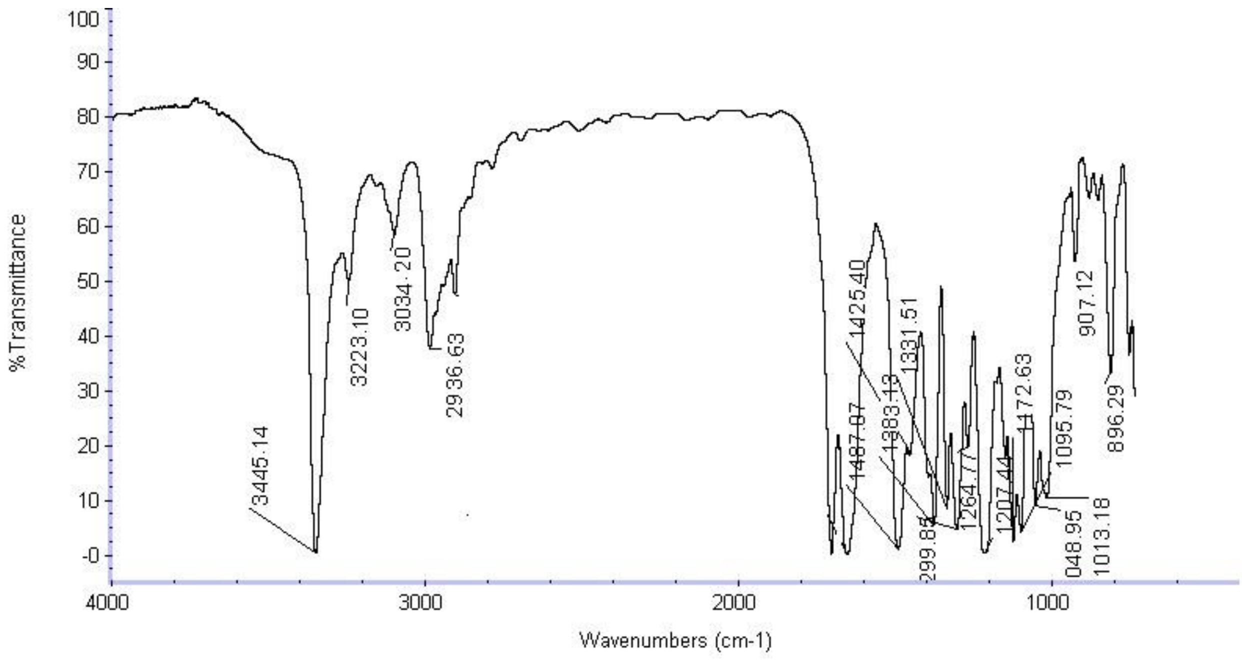
%Transmittance
100
90
80
70
60
50
40
30
20
10
-0
4000
3000
2000
1000
Wavenumbers (cm-1)
3445.14
3223.10
3034.20
2936.63
1425.40
1487.07
1383.13
1331.51
299.85
1264.77
1207.44
1172.63
1095.79
048.95
1013.18
907.12
896.29

Compound-1j

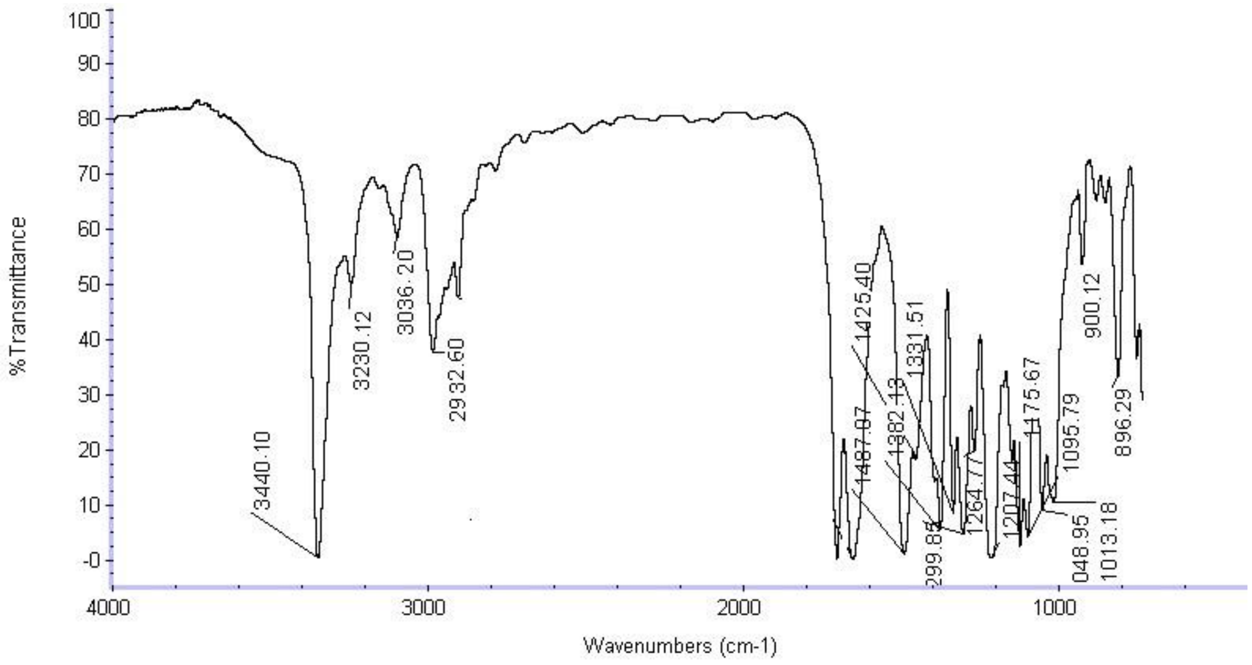
%Transmittance
100
90
80
70
60
50
40
30
20
10
-0
4000
3000
2000
1000
Wavenumbers (cm-1)
3440.10
3230.12
3036.20
2932.60
1425.40
1487.07
1382.13
1331.51
299.86
1264.77
1207.44
1175.67
1095.79
048.95
1013.18
900.12
896.29

Compound- 1a

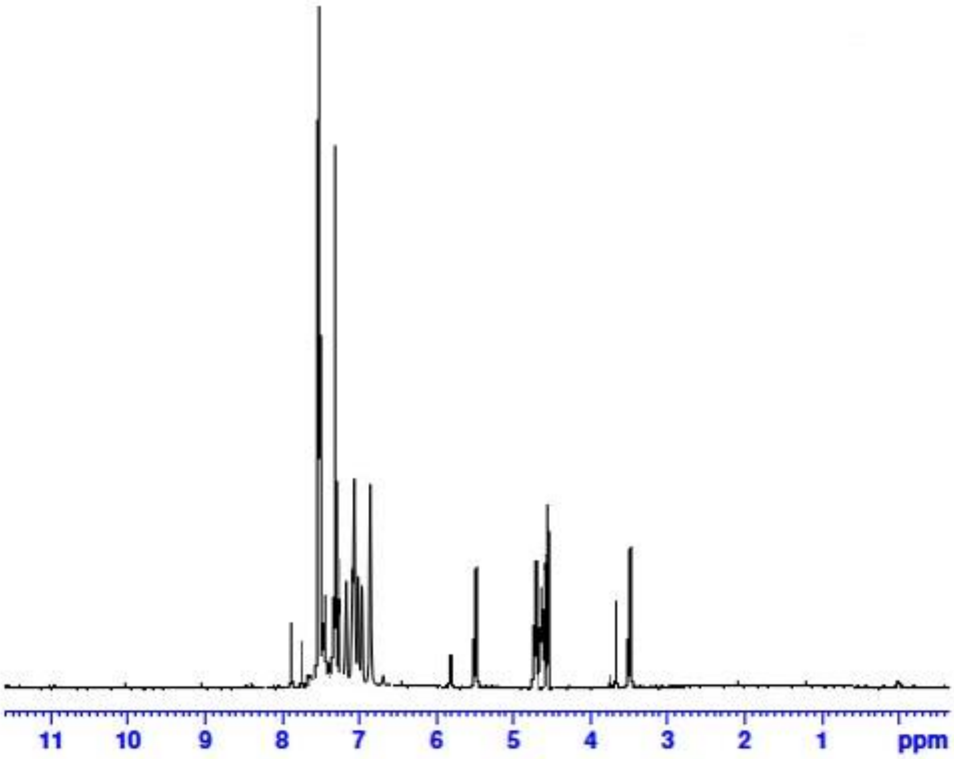

Compound-1b

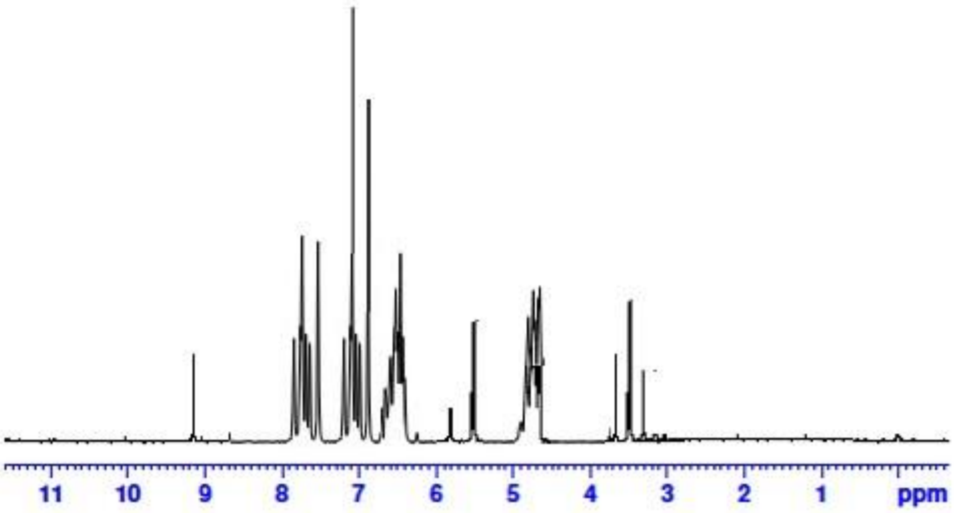

Compound-1c

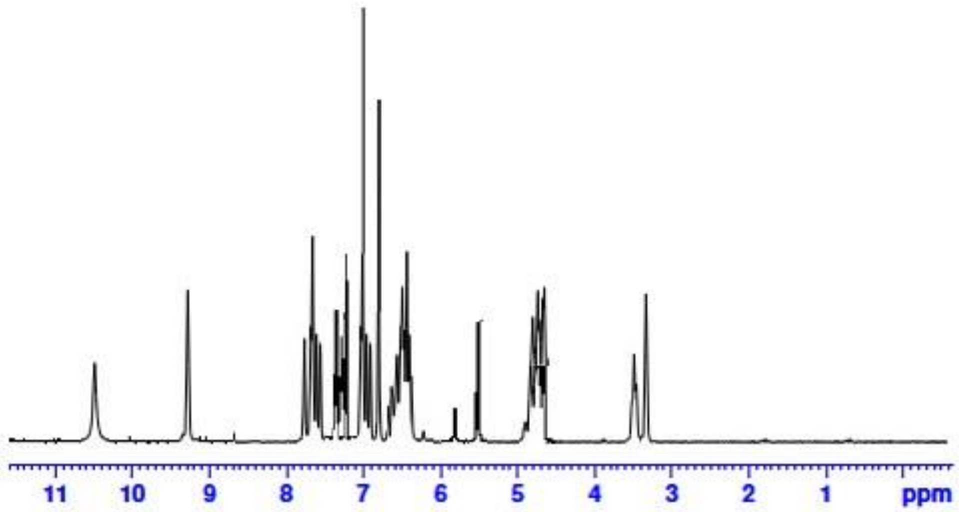

Compound-1d

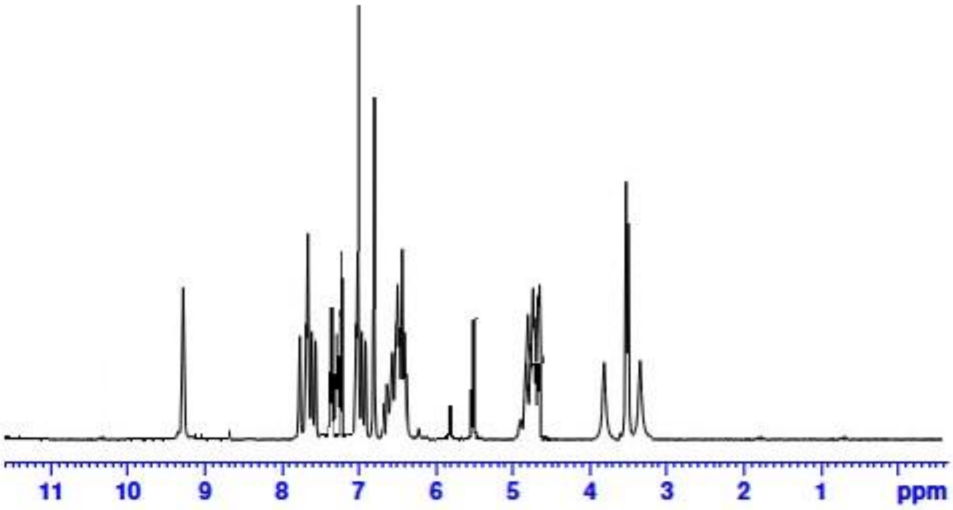

Compound-1e

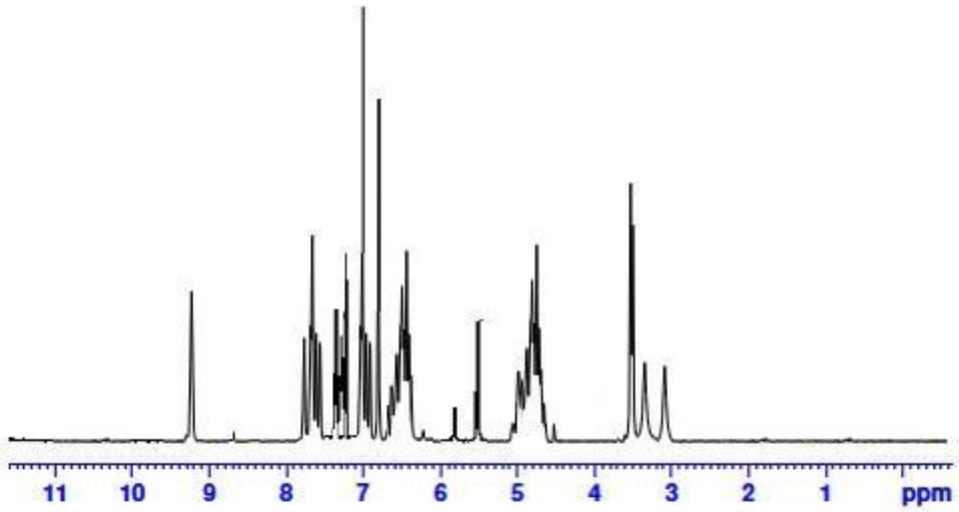

11
10
9
8
7
6
5
4
3
2
1
ppm

Compound-1f

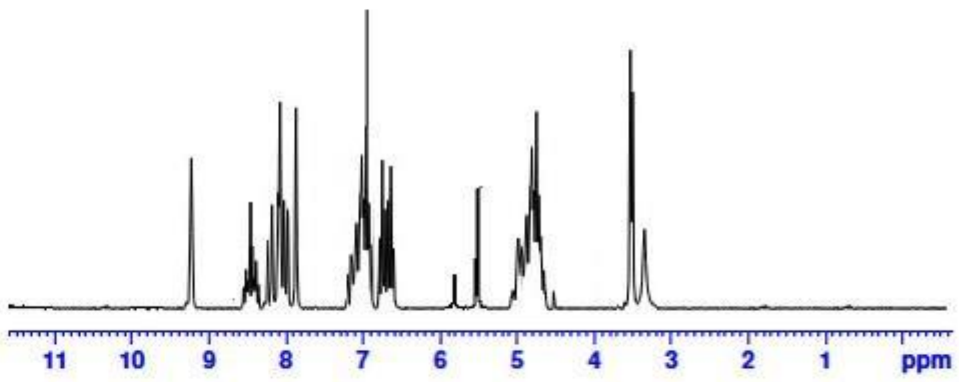

Compound-1g

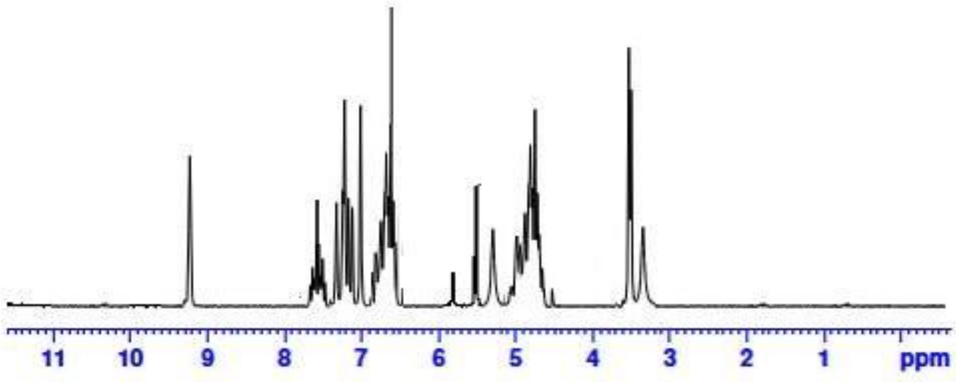

Compound-1h

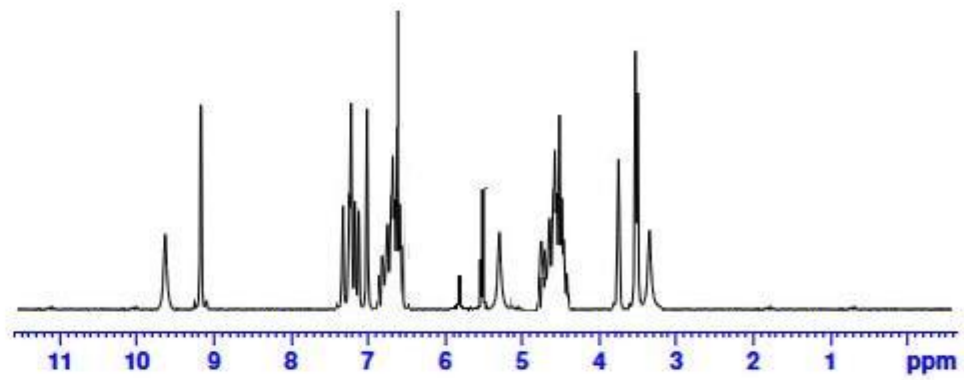

Compound-1i

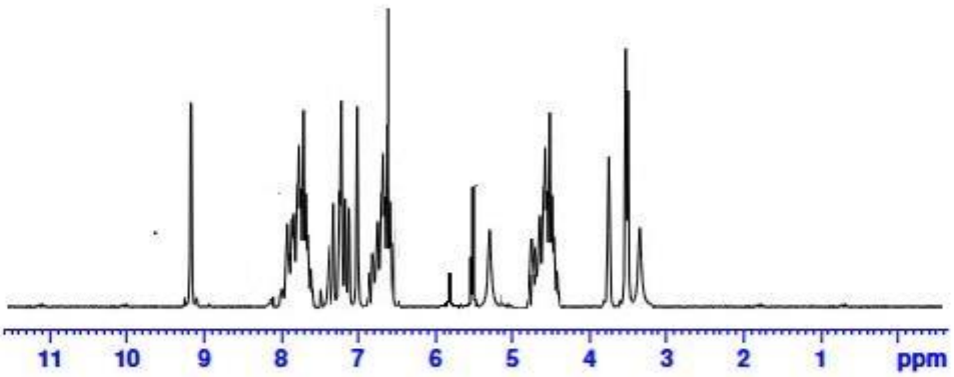

Compound-1j

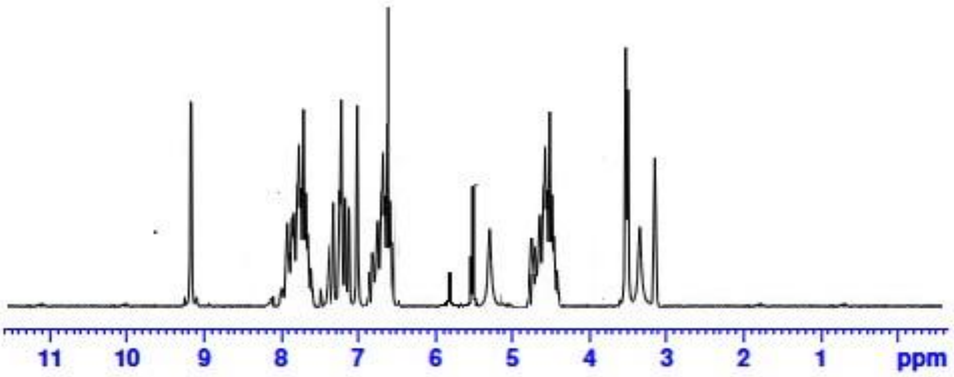

^{13}C-NMR spectra of Compound-1a

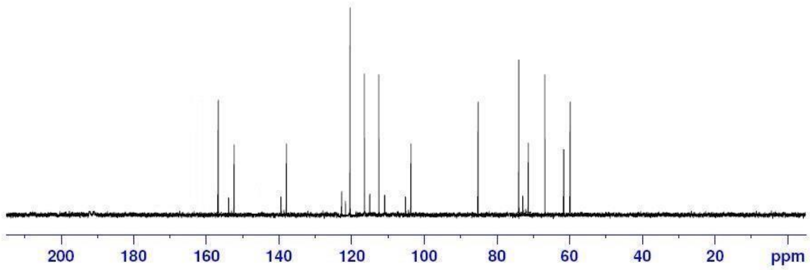

Compound-1b

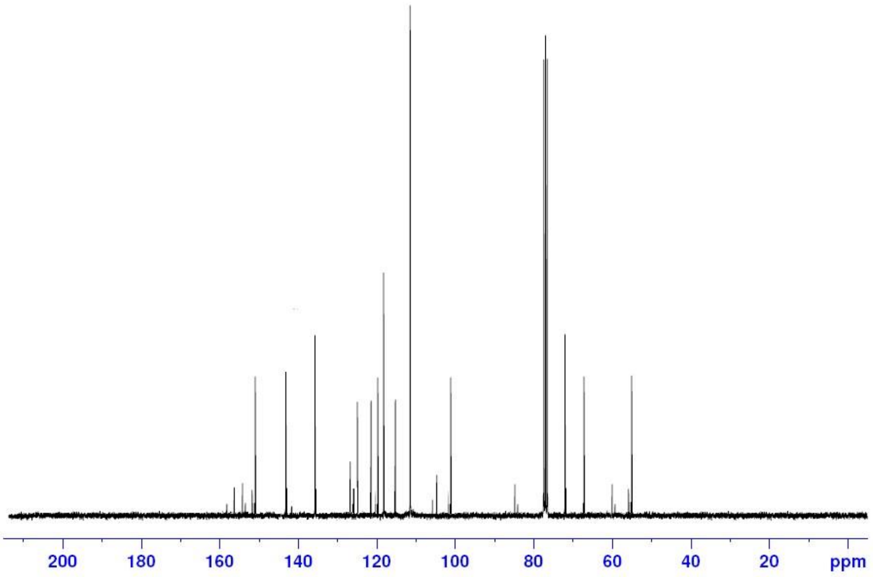
200
180
160
140
120
100
80
60
40
20
ppm

Compound 1c

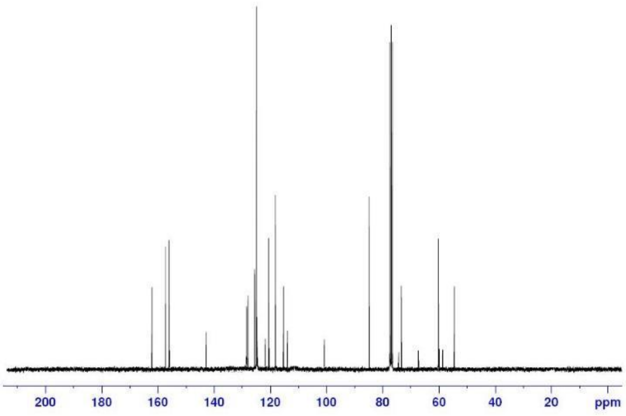
200
180
160
140
120
100
80
60
40
20
ppm

Compound 1d

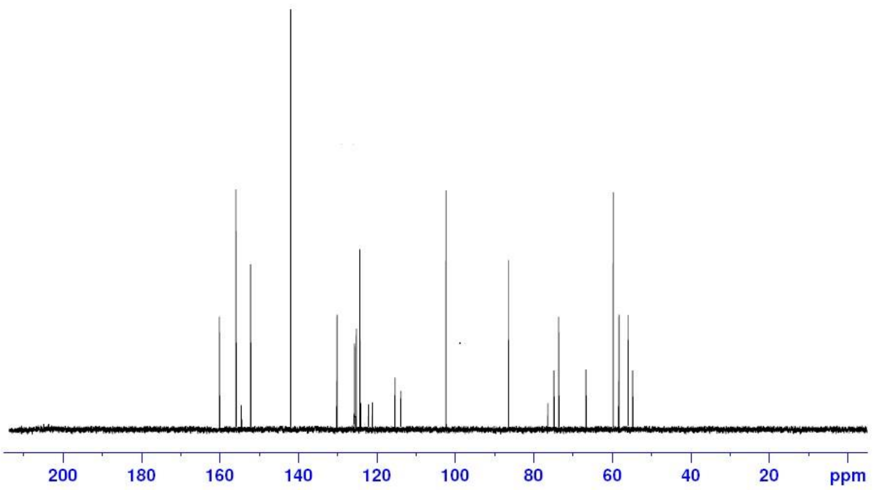

Compound 1e

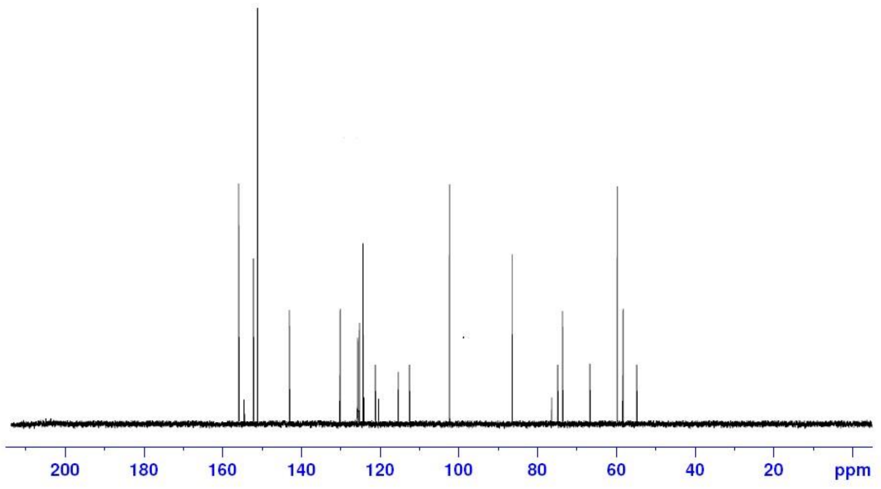

Compound 1f

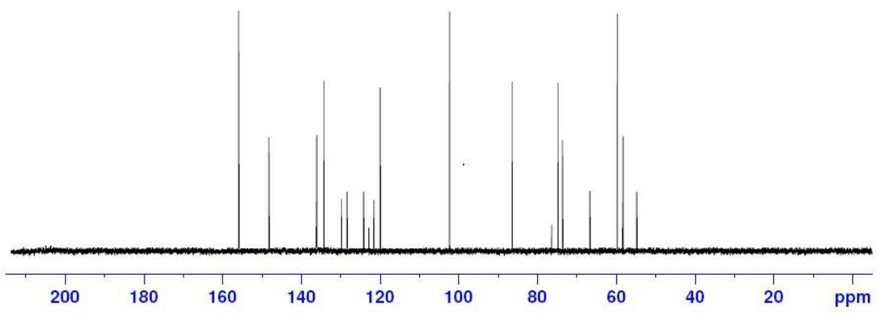

Compound 1g

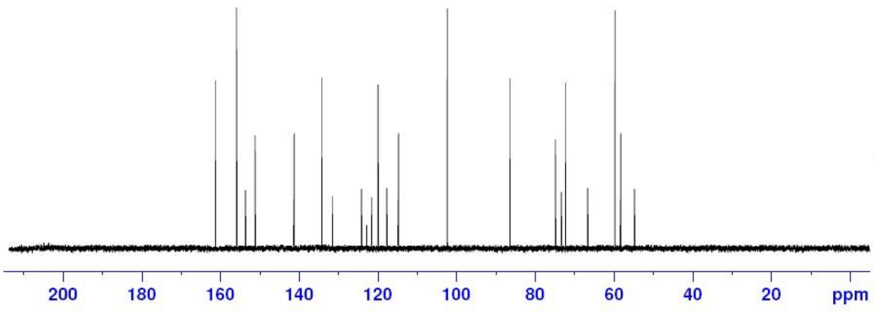

Compound 1h

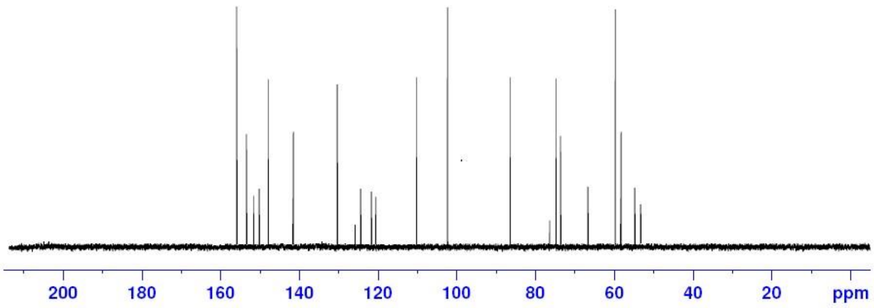

Compound 1i

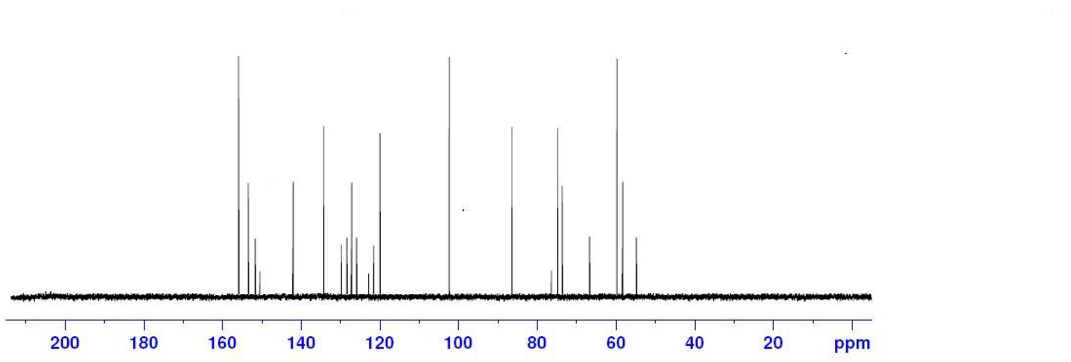
200
180
160
140
120
100
80
60
40
20
ppm

Compound 1j

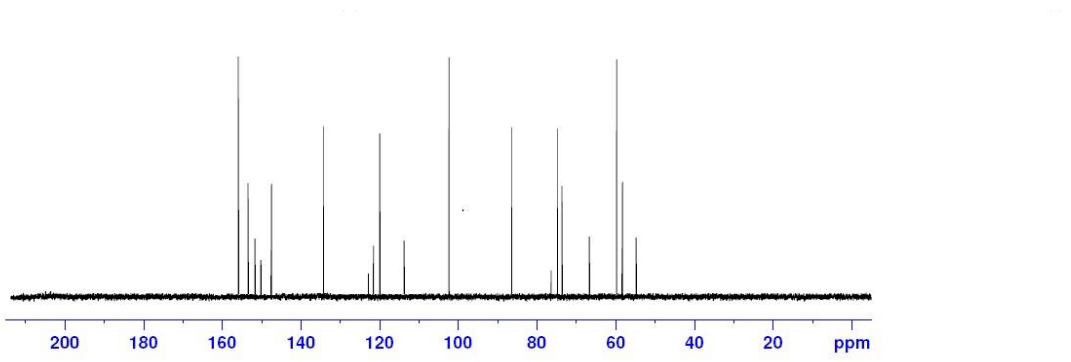
200
180
160
140
120
100
80
60
40
20
ppm
